# A novel CREB5/TOP1MT axis confers cisplatin resistance through inhibiting mitochondrial apoptosis in head and neck squamous cell carcinoma

**DOI:** 10.1186/s12916-022-02409-x

**Published:** 2022-07-01

**Authors:** Tong Tong, Xing Qin, Yingying Jiang, Haiyan Guo, Xiaoning Wang, Yan Li, Fei Xie, Hao Lu, Peisong Zhai, Hailong Ma, Jianjun Zhang

**Affiliations:** 1 Department of Oral and Maxillofacial-Head & Neck Oncology, Shanghai Ninth People’s Hospital, Shanghai Jiao Tong University School of Medicine; College of Stomatology, Shanghai Jiao Tong University; National Center for Stomatology; National Clinical Research Center for Oral Diseases; Shanghai Key Laboratory of Stomatology, No. 639, Zhizaoju Rd, Shanghai, 200011 People’s Republic of China; 2 Department of Oral and Maxillofacial Surgery, Shanghai Stomatological Hospital & School of Stomatology, Fudan University, Shanghai, 200001 People’s Republic of China; 3 Shanghai Key Laboratory of Craniomaxillofacial Development and Diseases, Fudan University, Shanghai, 200002 People’s Republic of China; 4 Department of Dentistry, Affiliated Hospital of Weifang Medical University, Weifang, 261000 People’s Republic of China; 5 Department of Clinical Laboratory, Ninth People’s Hospital, Shanghai Jiao Tong University School of Medicine, Shanghai, 200011 People’s Republic of China; 6 Department of Oral Pathology, Ninth People’s Hospital, Shanghai Jiao Tong University School of Medicine, Shanghai, 200011 People’s Republic of China; 7 Shanghai Institute of Immunology Center for Microbiota & Immune Related Diseases, Institute of Translational Medicine, Shanghai General Hospital, Shanghai Jiao Tong University School of Medicine, Shanghai, 200080 People’s Republic of China

**Keywords:** HNSCC, Cisplatin resistance, Mitochondrial apoptosis, CREB5, TOP1MT

## Abstract

**Background:**

Cisplatin resistance is one of the main causes of treatment failure and death in head and neck squamous cell carcinoma (HNSCC). A more comprehensive understanding of the cisplatin resistance mechanism and the development of effective treatment strategies are urgent.

**Methods:**

RNA sequencing, RT-PCR, and immunoblotting were used to identify differentially expressed genes associated with cisplatin resistance. Gain- and loss-of-function experiments were performed to detect the effect of CREB5 on cisplatin resistance and mitochondrial apoptosis in HNSCC. Chromatin immunoprecipitation (ChIP) assay, dual-luciferase reporter assay, and immunoblotting experiments were performed to explore the underlying mechanisms of CREB5.

**Results:**

CREB5 was significantly upregulated in cisplatin-resistant HNSCC (CR-HNSCC) patients, which was correlated with poor prognosis. CREB5 overexpression strikingly facilitated the cisplatin resistance of HNSCC cells in vitro and in vivo, while CREB5 knockdown enhanced cisplatin sensitivity in CR-HNSCC cells. Interestingly, the activation of AKT signaling induced by cisplatin promoted nucleus translocation of CREB5 in CR-HNSCC cells. Furthermore, CREB5 transcriptionally activated TOP1MT expression depending on the canonical motif. Moreover, CREB5 silencing could trigger mitochondrial apoptosis and overcome cisplatin resistance in CR-HNSCC cells, which could be reversed by TOP1MT overexpression. Additionally, double-targeting of CREB5 and TOP1MT could combat cisplatin resistance of HNSCC in vivo.

**Conclusions:**

Our findings reveal a novel CREB5/TOP1MT axis conferring cisplatin resistance in HNSCC, which provides a new basis to develop effective strategies for overcoming cisplatin resistance.

**Supplementary Information:**

The online version contains supplementary material available at 10.1186/s12916-022-02409-x.

## Background

Head and neck squamous cell carcinoma (HNSCC) is a common cancer with a high morbidity, high mortality rate, and poor prognosis. Comprehensive therapies including surgery, chemotherapy, and radiotherapy are the main treatment regimens for HNSCC [1]. Chemotherapy based on cisplatin is currently one of the most important adjuvant treatments for locally advanced/metastatic HNSCC, which is essential to improve the survival rate of patients. Cisplatin has an irreplaceable position as the first-line treatment of HNSCC. However, inherent and acquired cisplatin resistance can directly lead to treatment failure, relapse, and even death in patients [1]. Once relapse, patients develop faster progression, cachexia, and multiple organ failure. Therefore, a thorough study of the mechanisms underlying cisplatin resistance is of great significance for exploring potential targets to reverse resistance and improve clinical efficacy.

In recent years, several mechanisms for cisplatin resistance have been developed, such as the reduction of cisplatin accumulation in cells [2], increased DNA repair in cancer cells [3], sulfhydryl-containing molecules binding to and inactivating cisplatin [4], and alterations in PIK3/AKT, JNK [5], p53, and the anti-apoptosis Bcl-2 family [6]. However, the regulation of apoptosis occurs in all of these diverse mechanisms. These apoptotic processes can be controlled by the balance between anti-apoptotic proteins Bcl-2 and Bcl-xL and the pro-apoptotic protein Bax. Mitochondria are the main site for the regulation of apoptosis in the Bcl-2 family [7]. The pro-apoptotic proteins lead to the increase of the permeability of the mitochondrial membrane and the release of cytochrome c, thereby activating caspase cleavage and apoptosis [8]. This mitochondrial-dependent mechanism of caspase activation is called the “intrinsic” pathway or mitochondrial pathway of apoptosis. These studies have fully demonstrated that mitochondrial apoptosis plays a key role in cisplatin resistance. However, treating cisplatin resistance in clinical practice remains extremely difficult. Therefore, clarifying the regulatory mechanism of mitochondrial apoptosis is still urgent for overcoming cisplatin resistance.

cAMP response element-binding 5 (CREB5), also known as CRE-BPA, is a member of the cAMP response element-binding (CREB) protein family. CREB is an important transcription factor that plays important roles in gene regulation, cell proliferation, and apoptosis [9–11]. In addition, CREB5 could promote the ability of invasion and metastasis in colorectal cancer cells [12]. Importantly, CREB can regulate mitochondrial gene expression and activate mitochondrial biogenesis [13–15]. Accumulating evidences have shown that transcription factors have potential therapeutic efficacy in the treatment of cancers. It has been reported that CREB5 could act as a powerful and independent predictor of overall poor survival for ovarian cancer patients [16]. In addition, CREB5 was shown to be a modulator of androgen receptor signals in prostate cancer cells, which can promote enzalutamide resistance in vivo and in vitro [17]. However, whether CREB5 has an effect on mitochondrial biogenesis and participates in cisplatin resistance is still unclear.

The purpose of this study is to explore whether CREB5 is involved in cisplatin resistance and its underlying molecular mechanism. In the study, we found that CREB5 regulated mitochondrial apoptosis through transcriptional activation of TOP1MT and participated in HNSCC cisplatin resistance. This newly revealed CREB5/TOP1MT signal axis has provided a novel target for cisplatin resistance in HNSCC, which is of great significance for overcoming resistance and improving clinical efficacy.

## Methods

### Ethical approval

The Ethics Committee of Shanghai Jiao Tong University approved our study. All experimental methods comply with the Declaration of Helsinki. All animal studies were conducted in accordance with the “Guidelines for the Care and Use of Laboratory Animals of the National Institutes of Health” and were approved by the Animal Care and Use Committee of Shanghai Jiao Tong University. The experimental mice were kept in the Central Laboratory Animal Facility of the Ninth People's Hospital Affiliated to Shanghai Jiao Tong University School of Medicine.

### Patients and specimens

All the samples were collected from the Department of Oral and Maxillofacial-Head and Neck Oncology, Ninth People’s Hospital, Shanghai Jiao Tong University School of Medicine (Shanghai, China). After tissues were collected from the human body, they were quickly transferred to a tissue preservation solution for storage. RNA was extracted before tissues were frozen. Because the tissue was not repeatedly frozen and thawed, the experimental data accurately reflected the level of RNA in the body.

Our research involving human participants, human material, or human data has been performed in accordance with the Declaration of Helsinki and approved by the ethics committee of Ninth People’s Hospital Affiliated to Shanghai Jiao Tong University School of Medicine (the reference number: SH9H-2021-TK57-1).

### Cell cultures

The human HNSCC cell lines HN4 and HN30 were kindly provided by the University of Maryland Dental School, USA. All of the above cells were maintained in DMEM with 10% FBS and cultured at 37°C with 5% CO_2_. CR-HNSCC cells (HN4/DDP and HN30/DDP) were constructed from HN4 and HN30 through a gradient increasing the cisplatin dose and maintained in the culture medium containing 20 μM cisplatin (Howson, China).

### Agilent expression profile chip experiment

The Agilent SurePrint G3 Human Gene Expression v3 8x60K Microarray (DesignID:072363) chip experiment and the data analysis of four samples were conducted at OE Biotechnology Co., Ltd., (Shanghai, China). The Kyoto Encyclopedia of Genes and Genomes (KEGG) analysis was performed by DAVID (https://david.ncifcrf.gov).

### MTT assay

Cell viability was checked using an MTT assay. To determine the half maximal inhibitory concentration of cisplatin (IC50_cisplatin_) on HNSCC cells, the tumor cells were seeded in triplicate into 96-well plates with 3000 cells per well. Cells were incubated with serial doses of cisplatin at 24 h post-seeding. MTT (Sigma-Aldrich, USA) was added to each well (5 mg/ml) at 72 h post-treatment, then 150 μL of dimethyl sulfoxide (DMSO) was added to each well to dissolve the formazan formed after 4-h incubation. The absorbance at 490 nm was measured using a plate reader (SpectraMax i3x, Molecular Devices, USA).

To evaluate the cell proliferation ability, tumor cells were seeded in triplicate into 96-well plates with 1000 cells per well. During the cultivation, the growth of tumor cells was monitored by measuring the absorbance at 490 nm by daily MTT detection.

### Immunoblotting

Total proteins from the whole cell lysate were separated on 4–20% polyacrylamide gradient gel and transferred onto the PVDF membrane (Merck Millipore, USA). After being blocked with 5% skim milk, PVDF membranes were blotted with the primary antibody overnight at 4 °C. The GAPDH was used as a loading control. The signal was developed using an Odyssey Infrared Imaging System (Biosciences, USA) or with ECLUltra (New Cell and Molecular Biotech, Suzhou, China). All the antibodies are listed in Additional file 1: Table S1.

### RNA extraction and real-time PCR analysis

The total RNA from cultured cells or tissues was extracted using TRIzol reagent (Invitrogen, USA) and then reverse transcribed into cDNA using the Prime Script RT kit (Takara, Japan). Real-time PCR was performed using the Hieff UNICON® qPCR SYBR Green Master Mix kit (YEASEN, China) and the ABI StepOne real-time PCR system (Applied Biosystems, USA). β-actin was used as a housekeeping gene. The primer sequences are listed in Additional file 1: Table S2.

### Lentivirus transduction

CREB5 and TOP1MT overexpression lentivirus and control lentivirus were purchased from Hanyin Biotechnology Limited Company (Shanghai, China). Lentiviral transduction was performed according to the manufacturer’s instructions with polybrene (final concentration of 10 μg/ml). Puromycin (10 μg/ml) was used to select stable transduced cells at 72 h post-transduction. After continuous cultivation for 1 month, the corresponding experiment was carried out. CREB5 and TOP1MT gene overexpression cells and control cells were named CREB5, TOP1MT, and Vector, respectively.

### Small-interfering RNA (siRNA) transfection

Small-interfering RNA (siRNA, Sangon Biotech, China) was transfected using Lipofectamine 2000 reagent (Invitrogen, USA) at a final concentration of 50 nM. The siRNA sequences are listed in Additional file 1: Table S3.

### Colony formation assay

Around 1000 cells were seeded into six-well plates and cultured for 24 h, then the medium was replaced with fresh DMEM with or without cisplatin. The cells were cultured in the incubator for another 8–15 days until visible colonies were formed. The colonies were fixed and stained with crystal violet (YEASEN, China). The colony-forming ability was calculated based on the colony size and number.

### Cell apoptosis assay

Cells were incubated with or without cisplatin for 24 h. Then, the cells were removed from the culture using trypsin and washed twice with ice-cold PBS. Apoptotic cells were stained using APC Annexin V Apoptosis Detection Kit (BD Pharmingen^TM^, USA) and quantified by flow cytometry (BD Biosciences, USA).

### Metabolic flux assay

Cellular oxygen consumption rate (OCR) was analyzed on an XF96 Extracellular Flux Analyzer (Seahorse Bioscience, USA). The day before the experiment, 1 × 10^5^ cells/well were seeded on Seahorse 96-well culture plates and incubated overnight. The seahorse XF calibration solution was used to hydrate the probe overnight in a carbon dioxide-free incubator at 37 °C. On the day of the experiment, the detection solution was prepared with seahorse XF Base Medium .Take out Seahorse 96-well culture plates and observe the cell state and density under a microscope. Change the cell culture medium into the detection solution and place it in a carbon dioxide-free incubator at 37 ° C for 1h. Prepare and dilute the drug to the required concentration, and add it into the four dosing holes of ABCD on the test board according to the experimental design. For mitochondrial fitness tests, OCR was measured sequentially at basal, and following the addition of 1 μM oligomycin (O), 0.5 μM luoro-carbonyl cyanide phenylhydrazone (F), and 1 μM antimycin (A).

### Mitochondrial activity analysis

Cells were seeded onto six-well plates (10–20% confluence). About 48 h later, Mito-Tracker Red CMXRos (200 μM stock solution; Beyotime, China) was added to the culture medium (the final concentration was 20–200 nM) and incubated at 37°C for 15 min. Then, the supernatant was removed, a fresh cell culture medium (pre-warmed to 37°C) was added to the wells, and Hoechst 33342 (Beyotime China) was used to stain nuclei. Quantitative analysis of mitochondrial activity was performed using the Cytation™ 5 cell imaging multifunctional detection system (BioTek, USA).

### Measurement of ATP level

The Enhanced ATP Assay Kit (Beyotime, China) was used to determine the ATP level in cells. Cells were washed with ice-cold PBS and then lysed with 200 μl of lysis buffer for each well of the six-well plate. Scrap to lift the cells and the lysate was clarified by centrifugation (12,000×g at 4°C for 5 min). In a 96-well plate, 20 μl of lysate was added to each well, which contained 100 μl of ATP detection solution per well. The detection was performed by a multifunctional microplate reader (SpectraMax i3x, Molecular Devices, USA).

### Measurement of mitochondrial-ROS (MT-ROS) level

Mitochondrial-ROS (MT-ROS) was measured by the change in fluorescence of the MitoSOX Red Mitochondrial Superoxide Indicator (YEASEN, China). Briefly, cells were plated in six-well plates and cultured in a cell incubator for 24 h. MitoSOX Red Mitochondrial Superoxide Indicator was added at 5 μM after being washed in ice-PBS and incubated at 37 °C in the dark for 10 min. The detection was performed by a multifunctional microplate reader (SpectraMax i3x, Molecular Devices, USA) after being washed with preheating buffer for 3 times.

### Measurement of mitochondrial-ATP (MT-ATP) level

Mitochondrial-ATP (MT-ATP) fluorescent probe pCMV-Mito-AT1.03 (Beyotime, China) was transfected by Lipofectamine 2000 reagent (Invitrogen, USA) to detect the mitochondrial ATP level in cells. The detection was performed by a multifunctional microplate reader (SpectraMax i3x, Molecular Devices, USA).

### Analysis of mitochondrial membrane potential (ΔΨm)

An enhanced mitochondrial membrane potential assay kit with JC-1 (Beyotime, China) was used to analyze the mitochondrial membrane protein. Remove the culture supernatant of adherent cells and wash it with PBS. Then, 1×JC-1 working solution was added to the medium for 20 min at 37 °C in the dark to label the mitochondria. Wash twice with JC-1 dyeing buffer, and add 2 ml cell culture medium. The detection was performed by a multifunctional microplate reader (SpectraMax i3x, Molecular Devices, USA). Normal mitochondrial potential showed red fluorescence (aggregate JC-1), and damaged mitochondrial potential showed green fluorescence (monomeric JC-1).

### Chromatin immunoprecipitation (ChIP) assay

Cells were fixed in 37% formaldehyde at room temperature for 15 min, and then, the DNA was sheared to 500–1000 bp by sonication. The chromatin was immunoprecipitated with anti-CREB5 antibody and IgG negative control (ChIP Assay Kit, Beyotime, China; DNA Purification Kit, Beyotime, China). ChIP-PCR primers, 2000 bp upstream of the TOP1MT promoter region, were designed and synthesized by RiboBio, China. The purified chromatin was analyzed and quantified by real-time PCR (primers are listed in Additional file 1: Table S4).

### Dual-luciferase reporter assay

Luciferase reporter (200 ng/well) and Renilla luciferase vector (10 ng/well; pRL-CMV; Hanyin, China) were transfected into HEK293T or tumor cells, which had been transfected with CREB5 overexpressing plasmid, at 24 h post-seeding into a 24-well plate (3×10^4^ cells per well) using Lipofectamine^TM^ 2000 (Invitrogen, USA). The luciferase activity was measured using a Dual-Luciferase Reporter Assay kit (Beyotime, Shanghai, China) according to the manufacturer’s instructions at 24 h post-transfection.

### Nuclear and cytoplasmic protein extraction

The cells were treated with an AKT inhibitor (MK2206; final concentration 10 μM; Beyotime, China) for 24 h, and then, the nuclear protein and plasma protein were extracted using a Nuclear and Cytoplasmic Protein Extraction Kit (Beyotime, China).

### Animal experiments

The tumor xenograft experiment was used to identify the role of CREB5 in HNSCC cells in vivo. CREB5 overexpression plasmid transfected HN30 cells or parent control HN30 cells were injected into BALB/C athymic nude mice (4-week-old; 1×10^7^ cells plus Matrigel per injection). The mixture was injected subcutaneously into the buttocks of nude mice to establish a tumor-bearing model. The tumor volume and mouse body weight were measured every 3–4 days. The mice were intraperitoneally injected with cisplatin (4 mg/kg) every 6 days of four injections at 11 days post-cell injection. The mice were sacrificed and the tumors were removed for subsequent analysis at 31 days. The tumor growth curve was plotted using tumor volume.

To evaluate the effect of targeting CREB5 and TOP1MT individually or in combination in cisplatin resistance in vivo, the nude mice were divided into four groups (three mice per group): the siNC group, siTOP1MT group, siCREB5 group, and siTOP1MT+siCREB5 group. Thereafter, HN30/DDP cells (1 × 10^7^ tumor cells plus Matrigel per injection) were subcutaneously injected into the buttocks of mice. Beginning approximately 11 days later, the mice received four intraperitoneal injections once every 6 days, with each injection containing 4 mg/kg cisplatin. For the siTOP1MT group, siCREB5 group, and siTOP1MT+siCREB5 group, the corresponding cholesterol-modified siRNA (5 nmol per spot; RiboBio, China) was injected around the tumor at the indicated time. The mice were sacrificed on day 31 for further analysis.

The animal research was conducted in accordance with the Basel Declaration outlines fundamental principles and approved by the ethics committee of Ninth People’s Hospital Affiliated to Shanghai Jiao Tong University School of Medicine (the reference number: SH9H-2021-A896-1).

### Immunohistochemical (IHC) analysis

The xenografts were fixed, dehydrated, and embedded in paraffin. Then, the sections were deparaffinized, rehydrated, immersed in a citric acid buffer for heat-induced antigen retrieval, and then immersed in 0.3% hydrogen peroxide to block endogenous peroxidase activity, using 10% goat serum blocking agent and incubation with primary antibody overnight at 4°C. DAKO ChemMate Envision kit/HRP (Dako-Cytomation, USA) was then used for the development of sections, and the sections were counterstained with hematoxylin, dehydrated, removed, and fixed. Finally, the sections were observed under a microscope, and the tissues with brown staining on the cytoplasm, nucleus, or cell membrane were considered positive. A tissue microarray (TMA) was prepared using 70 HNSCC and 18 normal oral mucosal tissues obtained from the Ninth People’s Hospital Affiliated to Shanghai Jiao Tong University between 2007 and 2008, from patients who underwent surgery and were diagnosed through pathological examinations.

### Evaluation of the therapeutic value of siCREB5 and siTOP1MT via the patient-derived xenograft (PDX) model

This experiment was approved by the laboratory animal care and use committee of the hospital. The PDX model was constructed as previously reported (Zhou et al., 2019). The patient’s tumor tissue was cut into 20–30-mm^3^ pieces and planted on the flank of BALB/C nude mice (6 weeks, male, about 20g). When the tumor volume reached 1500–2000 mm^3^, the mice were sacrificed. Tumor tissues were cut into pieces and seeded again for passaging. Third-passage mice with tumor volumes of 150–250 mm^3^ were used to evaluate the therapeutic value of the siCREB5 and siTOP1MT. Tumor volume (TV) was recorded every 3–5 days, and mice were sacrificed after therapy for 4 weeks. The tumor growth inhibition rate (TGI) was calculated as

$$\mathrm{TGI}=\left(\text{TVvehicle}-\text{TVtreatment}\right)/\left(\text{TVvehicle}-\text{TVinitial}\right)\times100\%.$$

### Statistical analysis

The statistical analysis was performed using GraphPad Prism. The Student’s *t* test or one-way ANOVA was used to analyze the significance between two or more groups. The data were presented as the mean ± SEM of three independent experiments, where *p* < 0.05 was considered statistically significant (*p < 0.05, **p < 0.01, ***p < 0.001, ****p < 0.0001).

## Results

### CREB5 is highly expressed in cisplatin-resistant HNSCC (CR-HNSCC) cells and translocated into the nucleus by AKT phosphorylation

**Fig. 1:**
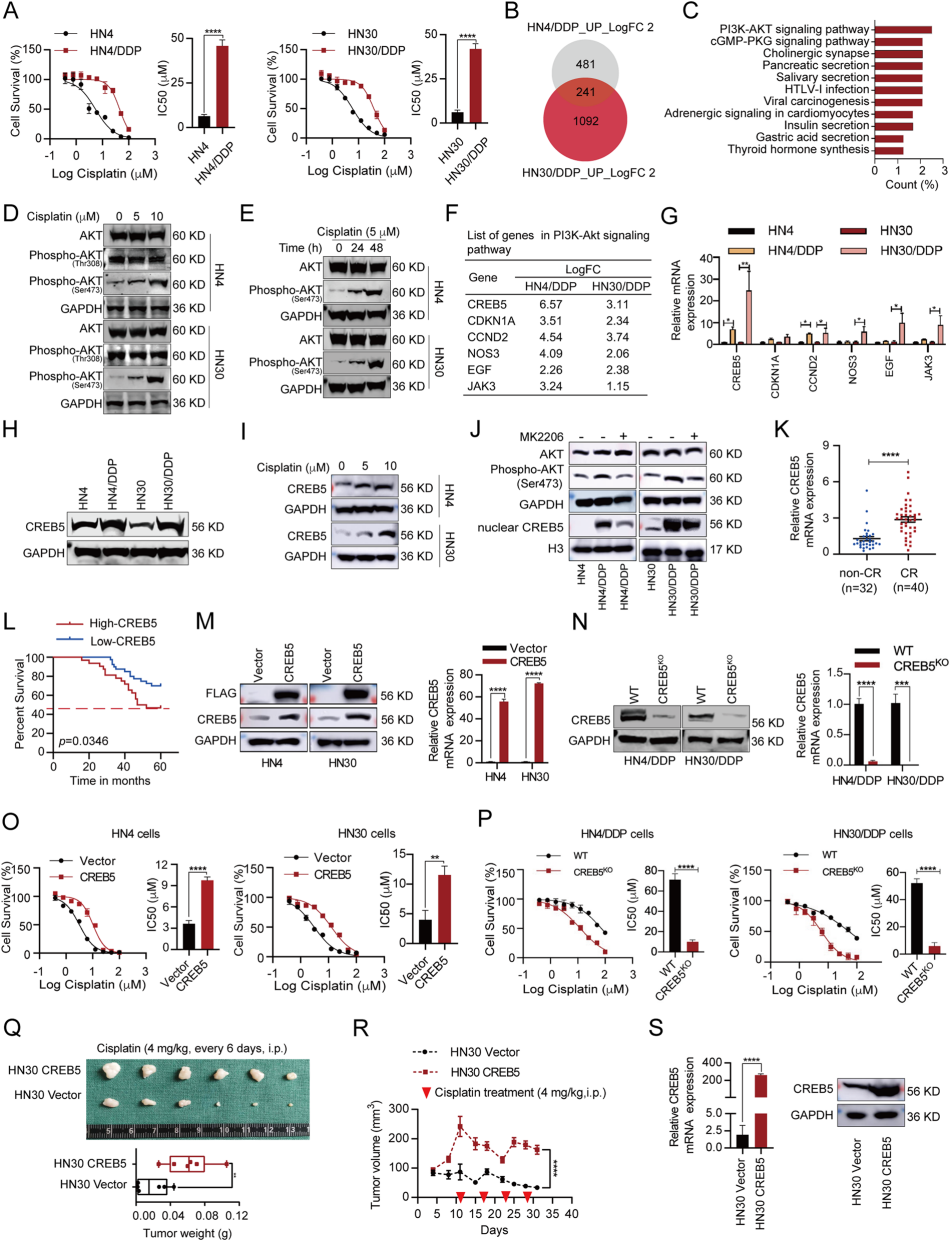
CREB5 is upregulated in cisplatin-resistant HNSCC cells and promotes cisplatin resistance. **A** MTT assay was used to analyze IC50_cisplatin_ in parent and cisplatin-resistant cells. **B** Venn diagram shows the expression of 241 genes upregulated simultaneously in HN4/DDP and HN30/DDP cells; the log FC (fold change) value is greater than 2. **C** Kyoto Encyclopedia of Genes and Genomes (KEGG) analysis of 241 genes upregulated in HN4/DDP and HN30/DDP cells compared to control cells. **D**, **E** Immunoblotting was used to detect the correlation of AKT phosphorylation level with cisplatin-stimulated concentration and time. **F** List of genes in PI3K-Akt signaling pathway. **G** RT-PCR analysis of the gene expression contained in the PI3K-Akt signaling pathway in parent and cisplatin-resistant cells. **H** Immunoblotting analysis of CREB5 expression in parent and cisplatin-resistant cells. **I** Immunoblotting was used to detect the correlation of CREB5 expression with cisplatin-stimulated concentration. **J** Immunoblotting analysis of phosphor-AKT (Ser 473) and nuclear CREB5 expression in parent cells and untreated or MK2206-treated (10 μM for 24 h) cisplatin-resistant cells. **K** RT-PCR analysis of CREB5 expression in 40 CR-HNSCC samples and 32 non-CR-HNSCC tissues. **L** Kaplan-Meier analysis of the correlation between CREB5 expression and overall survival. **M**, **N** RT-PCR and immunoblotting were used to detect the overexpression or knockout efficiency of CREB5. **O**, **P** MTT assay was used to analyze the correlation of IC50_cisplatin_ with CREB5 expression in parent and cisplatin-resistant cells. **Q** The tumor volume and weight in nude mice subcutaneously inoculated with un-transfected or CREB5-transfected HN30 cells at the end of the experiment are shown. **R** The tumor volume of control or CREB5-transfected HN30 cells was calculated every 3–4 days; cisplatin was injected intraperitoneally every 6 days for a total of four times. **S** Immunoblotting and RT-PCR analysis of CREB5 expression in subcutaneous tumor tissues of nude mice. *p < 0.05; **p < 0.01; ***p < 0.001; ****p < 0.0001

First, we constructed CR-HNSCC cell lines (HN4/DDP and HN30/DDP) by gradually increasing the concentration of cisplatin (Fig. 1A). To seek differential genes in the CR-HNSCC cells, RNA sequencing was performed. The results showed that 241 genes were upregulated in both HN4/DDP and HN30/DDP cells (Fig. 1B). KEGG was used to analyze 241 genes, and it was found that the PI3K-AKT signaling pathway ranked first (Fig. 1C). It was also observed that cisplatin treatment could significantly induce the phosphorylation of AKT at Ser 473 in a dose- and time-dependent manner (Fig. 1D, E and Additional file 1: Fig. S1A-C).

In addition, CREB5 expression had the greatest difference in CR-HNSCC cells in the PI3K-AKT signaling pathway (Fig. 1F, G). CREB5 was indeed highly expressed in CR-HNSCC cells in a cisplatin-concentration-dependent manner (Fig. 1H, I and Additional file 1: Fig. S1D, E). To clarify whether CREB5 was regulated by AKT phosphorylation and entered the nucleus as a transcription factor, we used the AKT inhibitor (MK2206) to detect the enrichment of nuclear CREB5 after inhibiting AKT activity. The results showed that the expression of phosphorylated AKT (ser 473) was upregulated and the nucleus translocation of CREB5 was increased in HN4/DDP and HN30/DDP cells. In addition, its nucleus translocation was significantly reduced after inhibiting the PI3K-AKT signaling pathway (Fig. 1J and Additional file 1: Fig. S1F, G) and downregulating AKT expression (Additional file 1: Fig. S2). The above data indicate that the transcription factor CREB5 is regulated by AKT phosphorylation and then translocated into the nucleus.

### CREB5 correlates with poor prognosis in HNSCC patients and confers cisplatin resistance to HNSCC cells

To further investigate the correlation between CREB5 expression and HNSCC cisplatin resistance, we used real-time PCR to quantify CREB5 mRNA levels in the extended HNSCC cohort. Consistent with the above data, CREB5 was upregulated in 40 CR-HNSCC samples compared with 32 non-CR-HNSCC samples (Fig. 1K). As shown in Table 1, high CREB5 expression was significantly correlated with larger tumor size (diameter greater than 4 cm) and advanced TNM (Tumor Node Metastasis) stage. Interestingly, patients with high CREB5 expression had poor responses to the TPF (docetaxel, cisplatin, 5-FU) strategy. In addition, COX regression analysis showed that CREB5 expression was an independent predictor for overall survival in HNSCC (Fig. 1L).

**Table 1:** Relationship between CREB5 mRNA level and clinicopathologic features (*N* = 72)

Characteristics	No. of patients		CREB5 2^-△△Ct^ Mean ± SEM	Non-parametric test value	*P* value
	No.	%			
Age (years)					
≥60	34	47.22%	2.18 ± 0.25	*Z* = *−0.073*	*0.942*
<60	38	52.78%	2.16 ± 0.24		
Gender					
Male	41	56.94%	2.19 ± 0.23	*Z = −0.034*	*0.973*
Female	31	43.06%	2.15 ± 0.27		
Smoking history					
Nonsmoker	51	70.83%	2.27 ± 0.21	*Z = −0.836*	*0.403*
Smoker	21	29.17%	1.93 ± 0.28		
Alcohol history					
Nondrinker	47	65.28%	2.05 ± 0.18	*Z = −0.396*	*0.692*
Drinker	25	34.72%	2.40 ± 0.36		
Tumor size (cm)					
≥4	26	36.11%	2.75 ± 0.33	*Z = −2.375*	*0.018*
<4	46	63.89%	1.84 ± 0.18		
Lymph node metastasis					
pN1 to pN2	42	58.33%	1.93 ± 0.19	*Z = −1.405*	*0.160*
pN0	30	41.67%	2.51 ± 0.31		
TNM stage					
I	18	25.00%	2.40 ± 0.29	*H = 11.917*	*0.008*
II	17	23.61%	1.36 ± 0.22		
III	19	26.39%	2.85 ± 0.35		
IV	18	25.00%	1.99 ± 0.39		
Pathological differentiation					
Well	32	44.44%	2.19 ± 0.27	*Z = −0.136*	*0.892*
Moderately/poorly	40	55.56%	2.15 ± 0.22		
Efficacy of TPF regimen					
Non-CR	32	44.44%	1.29 ± 0.18	*Z = −4.976*	*0.000*
CR	40	55.56%	2.88 ± 0.22		
Recurrence					
Yes	56	77.78%	2.27 ± 0.20	*Z = −0.935*	*0.350*
No	16	22.22%	1.82 ± 0.32		

*Abbreviations*: *CREB5* cAMP response element-binding 5, *SEM* standard error of mean, *CR* cisplatin resistance, *pN* pathological lymph node status, *TNM stage* tumor-lymph node-metastasis stage; 2^**-**△△Ct^ indicates the difference in the cycle number at which a sample’s fluorescent signal passes a given threshold above baseline (Ct) derived from a specific gene compared with that of GAPDH in tumor tissues

To explore the biological function of CREB5 in the cisplatin resistance mechanism of HNSCC, we established cell lines with CREB5 overexpression or knockout (Fig. 1M, N). Ectopic expression of CREB5 notably elevated the IC50_cisplatin_ in HN4 cells (3.635 ± 0.2522 μM *vs* 9.757 ± 0.278 μM), so it is in HN30 cells (3.977 ± 0.921 μM *vs* 11.51 ± 0.8776 μM, Fig. 1O). In HN4/DDP cells, CREB5 knockout reduced the IC50_cisplatin_ from 71.23 ± 3.394 μM to 9.848 ± 1.231 μM (Fig. 1P). In HN30/DDP cells, CREB5 silencing decreased the IC50_cisplatin_ from 52.15 ± 1.832 μM to 5.935 ± 1.467 μM (Fig. 1P).

To verify whether CREB5 conferred cisplatin resistance in vivo, we subcutaneously injected HN30 expressing with or without CREB5 into nude mice to establish xenograft tumor models. Overexpression of CREB5 promoted tumor growth and resulted in greater tumor weight (Fig. 1Q, R). Moreover, we observed the upregulation of CREB5 mRNA and protein level in the CREB5-expressing group (Fig. 1S). In addition, the percentage of Ki67 staining was significantly increased in the CREB5 overexpression group compared to the control (Additional file 1: Fig. S3). These results indicated that CREB5 could promote the cisplatin resistance of HNSCC in vitro and in vivo.

### CREB5 promotes HNSCC proliferation and inhibits mitochondrial apoptosis

**Fig. 2:**
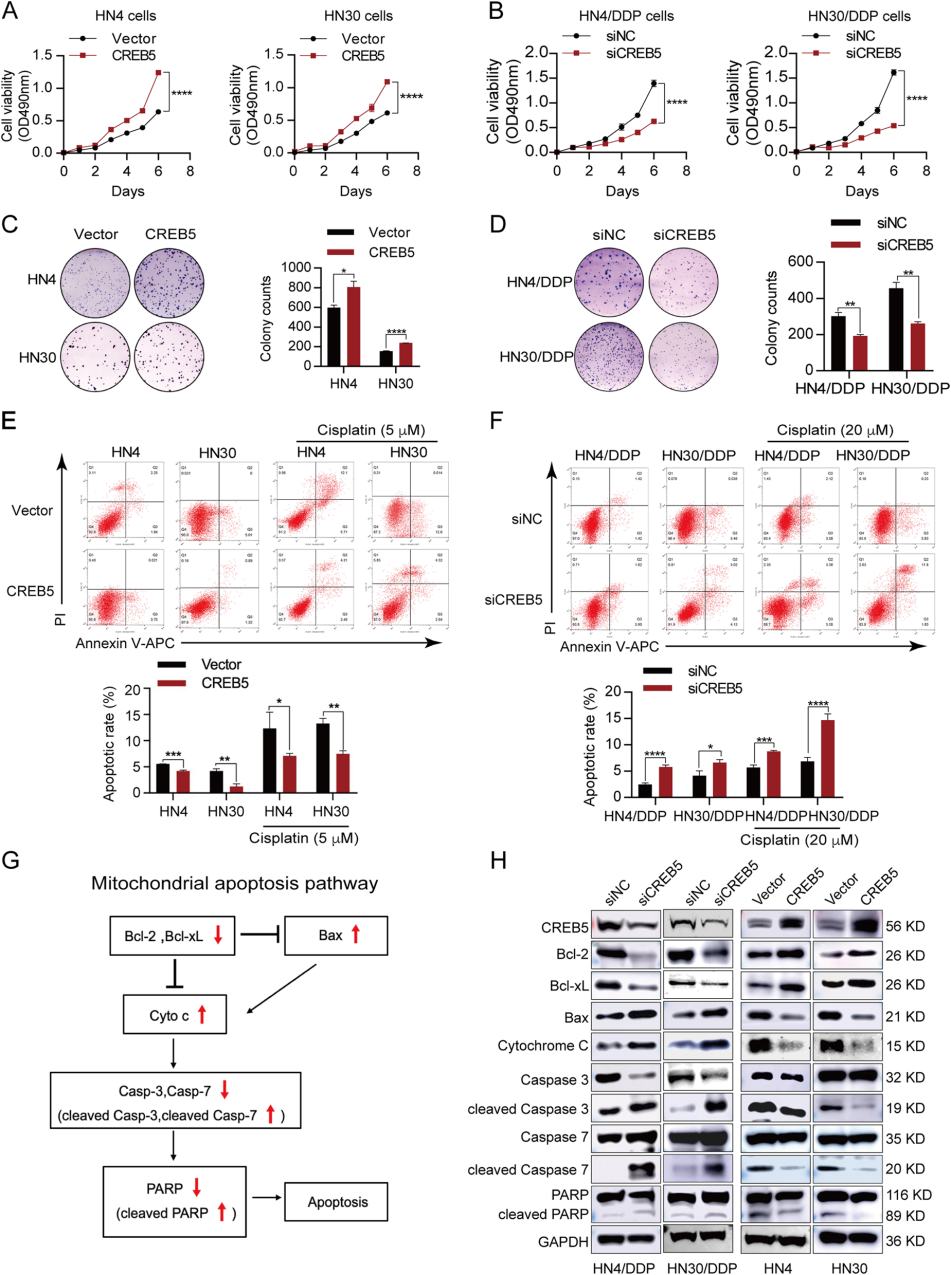
CREB5 regulates mitochondrial apoptosis in parent and cisplatin-resistant cells. **A**, **B** MTT assay was used to analyze the relationship between CREB5 expression and proliferation in parent and cisplatin-resistant cells. **C**, **D** The relationship between CREB5 expression and the ability to form colonies was analyzed in parent and cisplatin-resistant cells. **E** Flow cytometry was used to analyze the effect of CREB5 overexpression on the basal and cisplatin-induced (5 μM for 48 h) apoptosis percentage in HN4 and HN30 cells. **F** Flow cytometry was used to analyze the effect of CREB5 knockdown on the basal and cisplatin-induced (20 μM for 48 h) apoptosis percentage in HN4/DDP and HN30/DDP cells. **G** The flow chart of the mitochondrial apoptotic pathway. **H** Immunoblotting was used to analyze the effect of CREB5 knockdown or overexpression on the mitochondrial apoptosis pathway in cisplatin-resistant and parent cells, respectively. *p < 0.05; **p < 0.01; ***p < 0.001; ****p < 0.0001

The above results show that CREB5 promotes HNSCC cell resistance to cisplatin. Therefore, we next evaluated whether CREB5 affected cell proliferation, colony formation, and apoptosis. First, we determined the effect of CREB5 on cell proliferation and found that CREB5 promoted HN4 and HN30 cell proliferation (Fig. 2A), while CREB5 deletion significantly reduced HN4/DDP and HN30/DDP cell proliferation (Fig. 2B). We also observed that CREB5 improved the colony formation ability of HN4 and HN30 cells (Fig. 2C), while the number of colonies in HN4/DDP and HN30/DDP silencing CREB5 was greatly reduced (Fig. 2D). These results show that CREB5 can enhance HNSCC cell proliferation and colony formation ability.

To determine the role of CREB5 in HNSCC cell survival, a cell apoptosis assay was performed by flow cytometry. Compared with the vector group, the basal and cisplatin-induced apoptosis percentage of the CREB5 overexpression group was significantly reduced in HN4 and HN30 cells (Fig. 2E). Similarly, the knockdown of CREB5 greatly increased the basal and cisplatin-induced apoptosis percentage of HN4/DDP and HN30/DDP cells (Fig. 2F). To explore the mechanism, we detected the expression of mitochondrial apoptosis-related proteins (Fig. 2G). We found that overexpression of CREB5 could promote the expression of Bcl-2 and Bcl-xL and inhibit the expression of Bax and the release of cytochrome c, which inhibit the mitochondrial pathway of apoptosis and vice versa (Fig. 2H and Additional file 1: Fig. S4). Hence, we concluded that upregulation of CREB5 promoted tumor progression and inhibited mitochondrial apoptosis in HNSCC.

### CREB5 promotes transcription of the target gene-TOP1MT

**Fig. 3:**
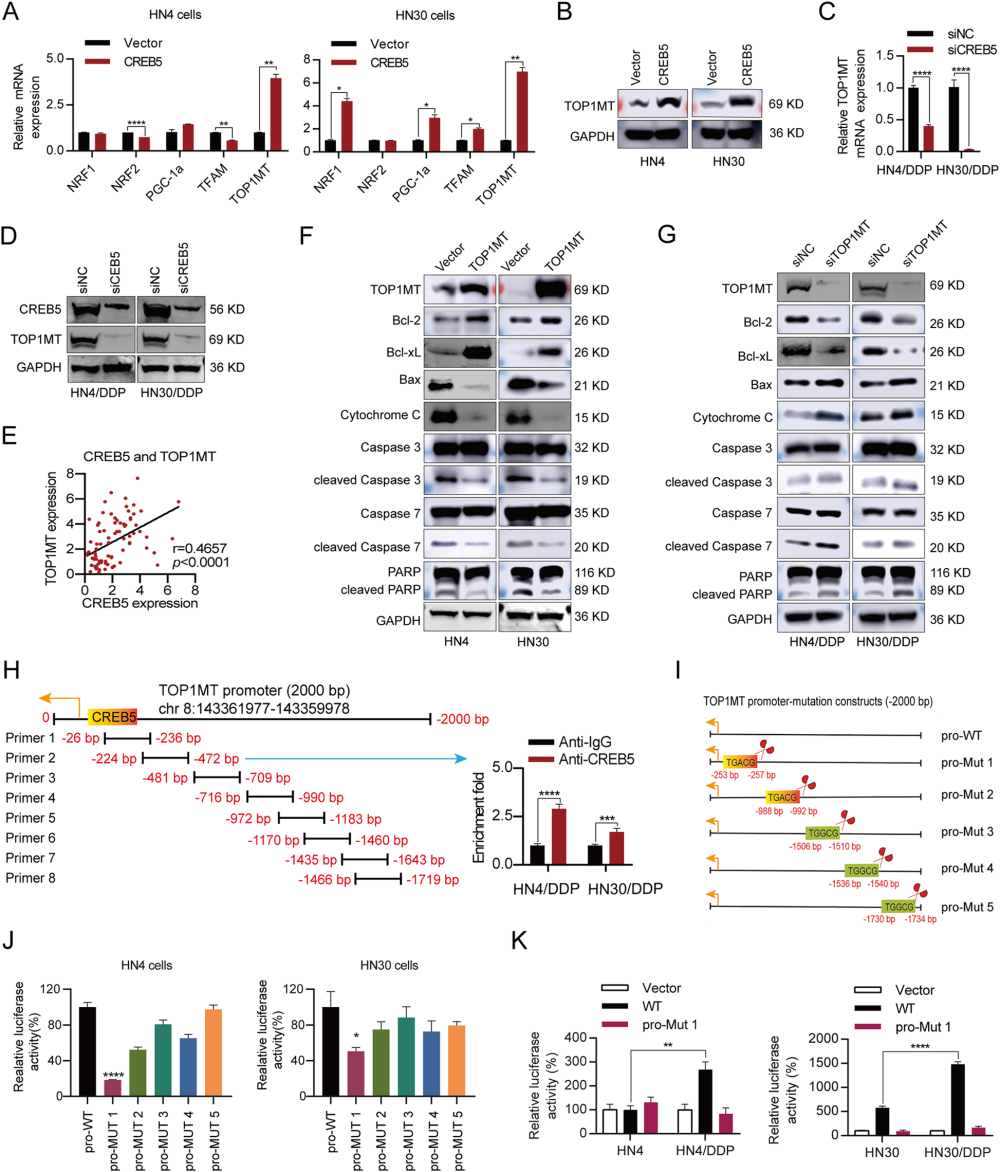
CREB5 promotes TOP1MT transcription in HNSCC. **A** RT-PCR was used to detect the effect of CREB5 on the expression of mitochondrial-related gene in HN4 and HN30 cells. **B** Immunoblotting was used to detect the effect of CREB5 on TOP1MT expression in HN4 and HN30 cells. **C**, **D** RT-PCR and immunoblotting were used to detect the effect of CREB5 knockdown on TOP1MT expression in HN4/DDP and HN30/DDP cells. **E** RT-PCR analysis of correlation of CREB5 expression with TOP1MT expression in HNSCC patients. **F**, **G** Immunoblotting was used to analyze the effect of TOP1MT on the mitochondrial apoptosis pathway in parent and cisplatin-resistant cells. **H** Schematic representation of TOP1MT promoter amplified by eight ChIP PCR primers. ChIP-PCR analysis of anti-CREB5- or IgG-immunoprecipitated TOP1MT promoter fragments extracted from HNSCC cells stably transfected with CREB5. **I** The luciferase deletion mutation vector of the predicted CREB5 target sequence (canonical motif: TGACG, non-canonical motif: TGGCG) in the TOP1MT gene. **J** Relative TOP1MT reporter activity in HN4 and HN30 cells co-transfected with CREB5 and luciferase reporter. **K** The effect of cisplatin resistance on TOP1MT reporter luciferase activity in HN4 and HN30 cells. *p < 0.05; **p < 0.01; ***p < 0.001; ****p < 0.0001

CREB, a key transcription factor family, plays an important role in maintaining cell survival by regulating the genes required to maintain mitochondrial function, autophagy, ROS production, and antioxidant response [18, 19]. There are several critical genes involved in mitochondrial biosynthesis, including peroxisome proliferator-activated receptor gamma coactivator 1-alpha (PGC-1α), nuclear respiratory factors (NRFs), mitochondrial transcription factor A (TFAM), mitochondrial DNA topoisomerase I (TOP1MT), peroxisome proliferator-activated receptor gamma (PPAR-γ), peroxisome proliferator-activated receptor gamma coactivator 1-beta (PGC-1β), mitochondrial transcription factor B1 (TFBM1), mitochondrial transcription factor B2 (TFBM2), transient receptor potential cation channel subfamily M member 2 (TRPM), and brain-derived neurotrophic factor (BDNF) [19–27]. To explore whether the above genes are transcriptional targets of CREB5, RT-PCR was performed and showed that only TOP1MT had consistent upregulation in HN4 and HN30 cells after CREB5 overexpression (Fig. 3A and Additional file 1: Fig. S5). TOP1MT plays an indispensable role in the modification of DNA topology, relieving tension and DNA supercoiling generated in the mitochondrial genome during replication and transcription [28]. Immunoblotting assay further confirmed that CREB5 overexpression could elevate the protein level of TOP1MT (Fig. 3B). Conversely, silencing CREB5 expression inhibited the mRNA and protein expression of TOP1MT in cisplatin-resistant cell lines (Fig. 3C, D). In addition, we choose CREB inhibitor (KG-501) and AKT inhibitor (MK2206) to detect further the relationship between CREB5, AKT, and TOP1MT. The result showed that the expression level of TOP1MT in cisplatin-resistant cells decreased after CREB5 was inhibited, while the combination with MK2206 resulted in a more significant decrease in TOP1MT expression (Additional file 1: Fig. S6). It is noteworthy that we found a positive correlation between CREB5 and TOP1MT expression in cisplatin-resistant HNSCC samples (Fig. 3E). Furthermore, TOP1MT overexpression promoted the expression of anti-apoptotic protein, including Bcl-2 and Bcl-xL, and inhibited the expression of pro-apoptotic protein, including Bax and cytochrome c (Fig. 3F and Additional file 1: Fig. S7A, B). In contrast, TOP1MT knockdown inhibited the anti-apoptotic protein and promoted pro-apoptotic protein in cisplatin-resistant cells (Fig. 3G and Additional file 1: Fig. S7C, D). This imbalance subsequently activated caspases 3 and 7 and PARP, initiating mitochondrial apoptosis. The results indicate that TOP1MT has a significant function in regulating the activation of the mitochondrial apoptosis pathway.

Given that CREB5 is a transcription factor, we speculate that CREB5 might promote the transcription of TOP1MT. We designed eight primers based on the transcription factor binding motif of CREB5 in the TOP1MT promoter region. ChIP-PCR results showed that only primer 2 could amplify the TOP1MT promoter fragment in HN4/DDP and HN30/DDP cells (Fig. 3H). The binding sites of CREB are divided into two types: the canonical motif (TGACG) and the non-canonical motif (TGGCG) [29]. There are two canonical motifs and three non-canonical motifs in the TOP1MT promoter region. To clarify the specific binding position of CREB5 in the TOP1MT promoter, we constructed five TOP1MT deletion mutation vectors (pro-Mut 1-5) based on known binding sites in the canonical and non-canonical motifs (Fig. 3I). A dual-luciferase reporter assay revealed that only the pro-Mut 1 vector had significantly lower luciferase activity than the wild-type (WT) in HN4 and HN30 cells (Fig. 3J). Moreover, TOP1MT promoter activation was significantly increased in cisplatin-resistant cells, which could be reduced by the deletion of −253 to −257 bp (Fig. 3K). Therefore, we concluded that the binding motif between CREB5 and the TOP1MT promoter was TGACG, which was located at ch8:143361725-143361721. To sum up, we conclude that TOP1MT participating in mitochondrial apoptosis was a novel transcriptional target of CREB5 in cisplatin-resistant cells.

### CREB5 regulates mitochondrial activity through TOP1MT in HNSCC

**Fig. 4:**
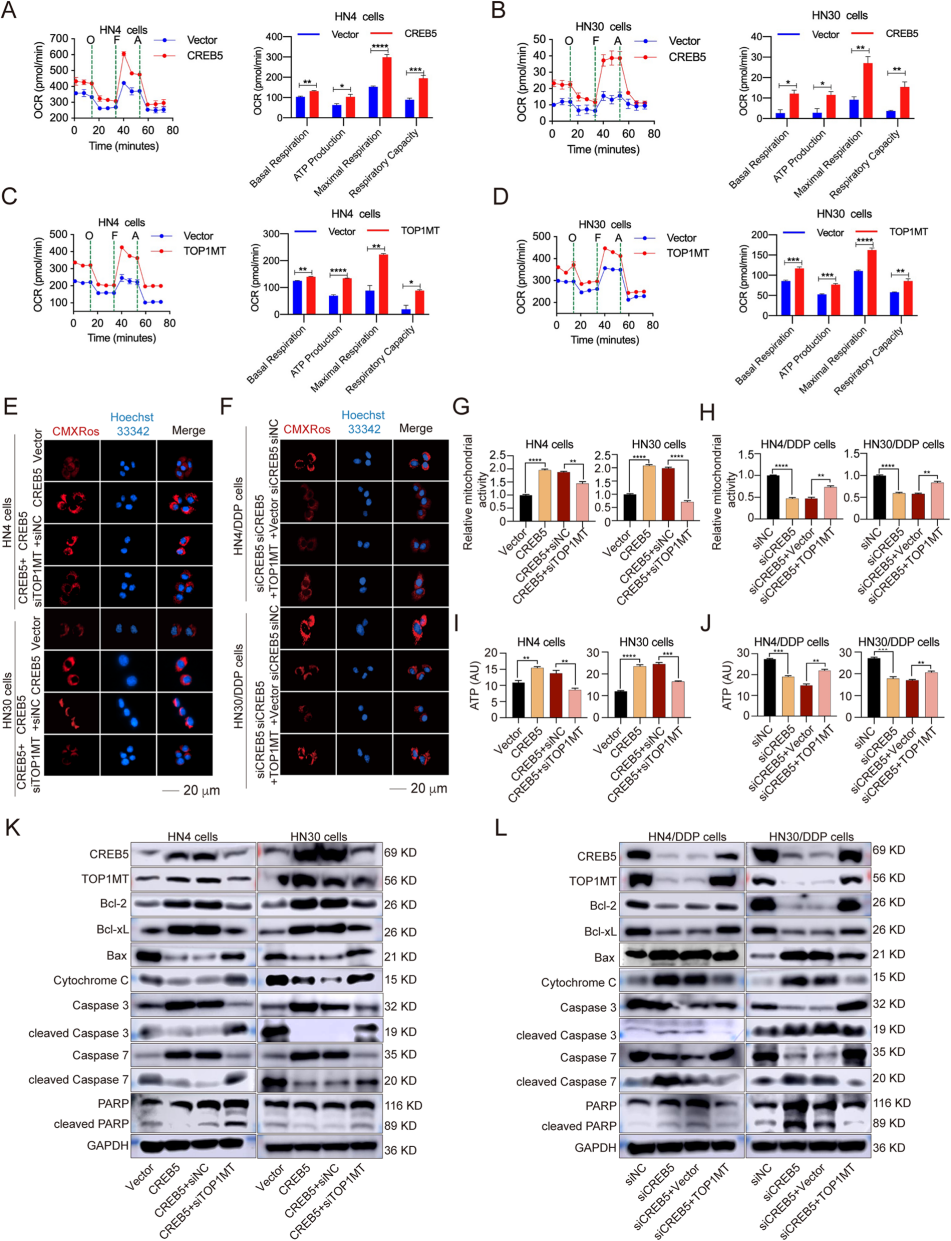
CREB5 regulates mitochondrial activity through TOP1MT. **A**–**D** Real-time OCR measurement of HN4 and HN30 cells treated as indicated. Cells were incubated with 1 μM oligomycin (O), 0.5 μM luoro-carbonyl cyanide phenylhydrazone (F), and 1 μM antimycin (A) for the indicated times. **E**, **F** Mito-Tracker Red CMXRos was used to detect the effect of CREB5 and TOP1MT expression on mitochondrial activity in parent and cisplatin-resistant cells. **G**, **H** Relative red fluorescence (Mito-Tracker Red CMXRos) values are shown. **I**, **J** The effect of CREB5 and TOP1MT expression on ATP levels was analyzed in HN4, HN30, HN4/DDP, and HN30/DDP cells. **K** Immunoblotting was used to detect the expression levels of CREB5, TOP1MT, Bcl-2, Bcl-xL, Bax, cytochrome c, caspase 3, cleaved caspase 3, caspase 7, cleaved caspase 7, PARP, cleaved PARP, and GAPDH in HN4 and HN30 CREB5 cells with TOP1MT knockdown. **L** Immunoblotting was used to detect the expression levels of CREB5, TOP1MT, Bcl-2, Bcl-xL, Bax, cytochrome c, caspase 3, cleaved caspase 3, caspase 7, cleaved caspase 7, PARP, cleaved PARP, and GAPDH in HN4/DDP and HN30/DDP siCREB5 cells with TOP1MT overexpression. *p < 0.05; **p < 0.01; ***p < 0.001; ****p < 0.0001

To explore the role of TOP1MT in CREB5 regulating mitochondrial activity, we examined the effects of CREB5 and TOP1MT on oxidative phosphorylation (OXPHOS). The results showed that both CREB5 or TOP1MT overexpression can promote the process of cellular OXPHOS. Further analysis found that basal respiration, ATP production, maximal respiration, and respiratory capacity were all increased after CREB5 and TOP1MT overexpression (Fig. 4A–D). Cellular oxygen consumption rate (OCR) gives a deeper idea of the mitochondrial activity and bioenergetic. These results demonstrated that both CREB5 and TOP1MT enhanced mitochondrial activity. In addition, Mito-Tracker Red CMXRos also was used to detect mitochondrial activity. The results show that overexpression of CREB5 enhances mitochondrial activity with an increasing red fluorescence intensity in HN4 and HN30 cells, which is reduced by TOP1MT knockdown. Conversely, CREB5 silencing inhibited mitochondrial activity, while the overexpression of TOP1MT reversed it in cisplatin-resistant cells (Fig. 4E–H).

Mitochondrial dysfunction leads to abnormal respiratory chain function and decreased ATP production, triggering mitochondrial apoptosis. We found that overexpression of CREB5 increased cellular ATP levels, which was reduced by TOP1MT knockdown in HN4 and HN30 cells (Fig. 4I). Furthermore, CREB5 silencing decreased ATP levels, while the overexpression of TOP1MT reversed that effect in cisplatin-resistant cells (Fig. 4J). The increasing red fluorescence intensity and ATP levels in cisplatin-resistant cells also proved that mitochondrial activity upregulation was a key event during cisplatin resistance (Additional file 1: Fig. S8). Besides, we detected the intracellular levels of mitochondrial superoxide (MT-SOX indicator), mitochondrial ATP (MT-ATP), and JC-1. The results showed that the levels of MT-ROS and MT-ATP were significantly increased after CREB5 expression upregulation, and the level of monomeric JC-1 was significantly decreased. On this basis, we found that the levels of MT-ROS and MT-ATP were correspondingly decreased after TOP1MT knockdown and the level of monomeric JC-1 was also increased (Additional file 1: Fig. S9). This is also consistent with our previous conclusion that CREB5 regulated mitochondrial activity through TOP1MT.

The overexpression of CREB5 inhibited mitochondrial apoptosis by increasing Bcl-2 and Bcl-xL as well as decreasing Bax, cytochrome c, cleaved caspase, and PARP. This effect was reversed by TOP1MT knockdown (Fig. 4K and Additional file 1: Fig. S10A, B). In contrast, in cisplatin-resistant cells, CREB5 silencing induced the mitochondrial apoptosis pathway, which was rescued by TOP1MT overexpression (Fig. 4L and Additional file 1: Fig. S10C, D). These results proved that CREB5 regulated mitochondrial apoptosis through TOP1MT, especially in cisplatin-resistant cells.

### CREB5 confers resistance to cisplatin through TOP1MT in HNSCC

**Fig. 5:**
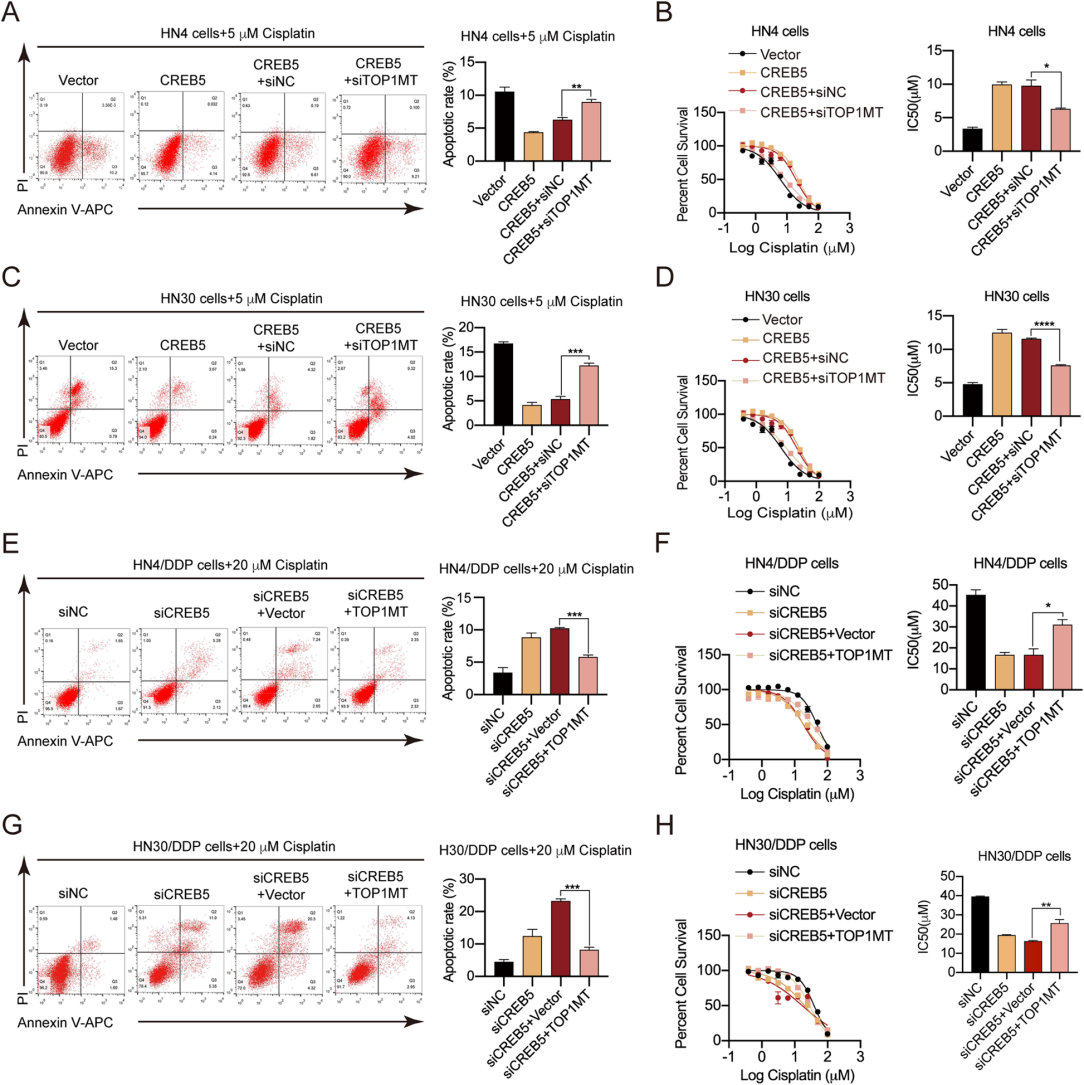
CREB5 inhibits apoptosis and promotes resistance to cisplatin by TOP1MT in HNSCC cells. **A** Flow cytometry analysis of cisplatin-induced (5 μM) apoptosis percentage in HN4 CREB5 cells with TOP1MT knockdown. **B** Percent cell survival was detected by MTT when TOP1MT was knocked down in HN4 CREB5 cells. IC50_cisplatin_ values are shown on the right. **C** Flow cytometry analysis of cisplatin-induced (5 μM) apoptosis percentage in HN30 CREB5 cells with TOP1MT knockdown. **D** Percent cell survival was detected by MTT when TOP1MT was knocked down in HN30 CREB5 cells. IC50_cisplatin_ values are shown on the right. **E** Flow cytometry analysis of cisplatin-induced (20 μM) apoptosis percentage in HN4/DDP siCREB5 cells with TOP1MT overexpression. **F** Percent cell survival was detected by MTT when TOP1MT was knocked down in HN4/DDP siCREB5 cells. IC50_cisplatin_ values are shown on the right. **G** Flow cytometry analysis of cisplatin-induced (20 μM) apoptosis percentage in HN30/DDP siCREB5 cells with TOP1MT overexpression. **H** Percent cell survival was detected by MTT when TOP1MT was overexpressed in HN30/DDP siCREB5 cells. IC50_cisplatin_ values are shown on the right. *p < 0.05; **p < 0.01; ***p < 0.001; ****p < 0.0001

According to the above findings, in which CREB5 regulated mitochondrial apoptosis through TOP1MT in cisplatin-resistant cells, we speculated that CREB5 might confer resistance to cisplatin through TOP1MT in HNSCC. Flow cytometry and an IC50 assay revealed that ectopic expression of CREB5 reduced the percentage of apoptotic cells and elevated the IC50_cisplatin_, while silencing TOP1MT increased apoptosis and decreased the IC50_cisplatin_ (Fig. 5A, B). We observed similar results in HN30 cell lines (Fig. 5C, D). In the resistant cell lines, CREB5 knockdown significantly increased the percentage of apoptotic cells and reduced the IC50_cisplatin_, while the overexpression of TOP1MT reversed that trend in HN4/DDP and HN30/DDP cell lines (Fig. 5E, H). The above results show that CREB5 confers cisplatin resistance through TOP1MT via apoptosis regulation in HNSCC.

### Double-targeting of CREB5 and TOP1MT overcomes cisplatin resistance in vivo

**Fig. 6:**
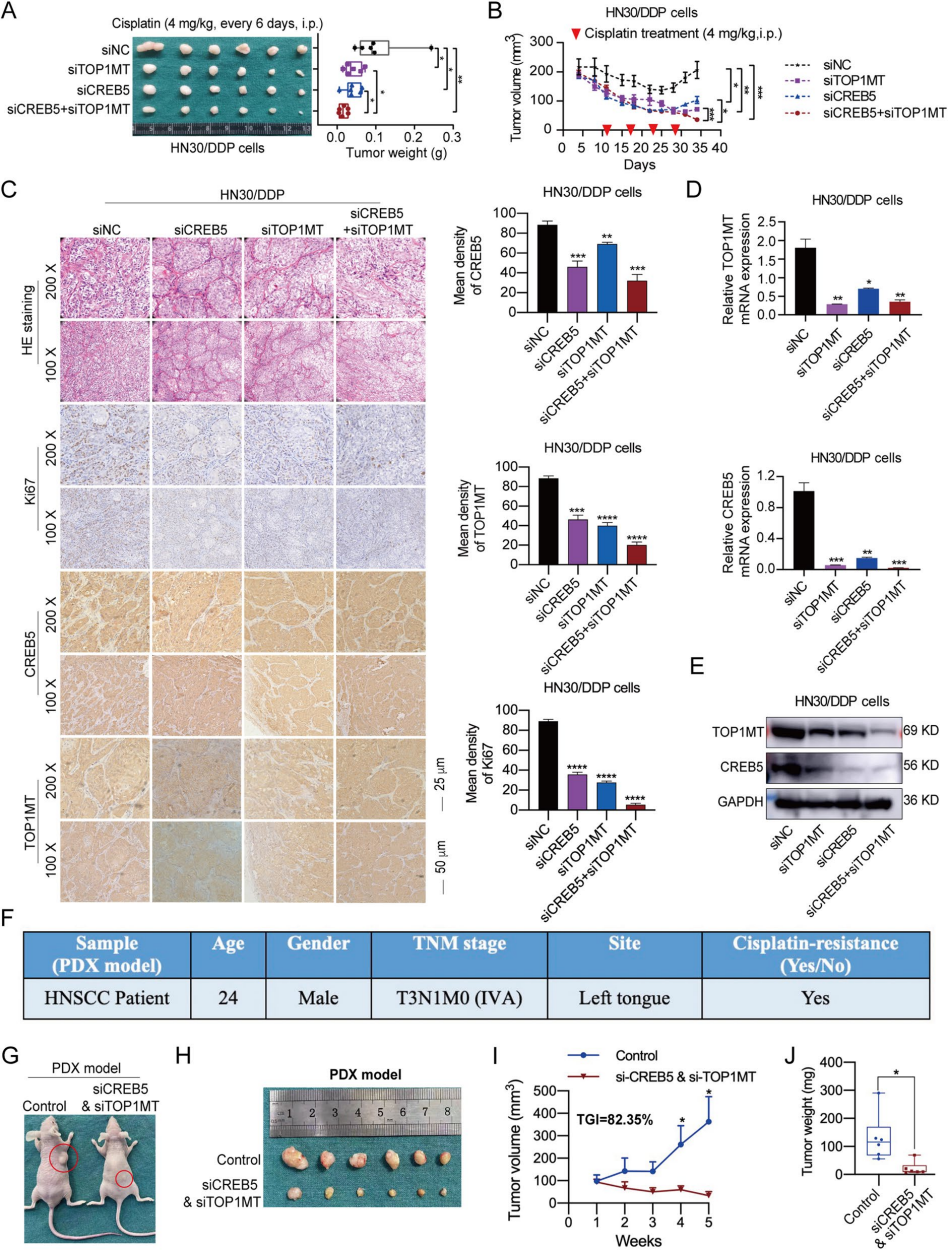
Double-targeting of CREB5 and TOP1MT has a potent antitumor effect in cisplatin-resistant HNSCC in vivo. **A** The tumor weight in nude mice subcutaneously inoculated with HN30/DDP cells treated with cholesterol-modified siCREB5 or/and siTOP1MT (5 μM) at the end of the experiment are shown. **B** The tumor volume of HN30/DDP cells treated with cholesterol-modified siCREB5 or/and siTOP1M (5 μM) was calculated every 3–4 days, and cisplatin was injected intraperitoneally every 6 days for a total of four times. **C** Ki67, TOP1MT, and CREB5 in subcutaneous tumor tissue of nude mice were detected and analyzed using IHC. **D**, **E** RT-PCR and immunoblotting analysis showed the expression of TOP1MT, CREB5, and GAPDH in the subcutaneous tumor tissue of nude mice. **F** Sample information of PDX model. **G**, **H** Image of mice and tumor after siCREB5 and siTOP1MT administration for 3 weeks. **I**, **J** Nude mice were used to construct the PDX model. The treatment group was given siCREB5 and siTOP1MT (5 μM) every day. The tumor volume and weight were recorded every week, and the mice were killed after 3 weeks. *p < 0.05; **p < 0.01; ***p < 0.001 ****p < 0.0001

To determine whether double-targeting of CREB5 and TOP1MT overcomes HNSCC resistance to cisplatin, we established a cisplatin-resistant xenograft model of HNSCC by subcutaneously injecting HN30/DDP cells into the buttocks of nude mice. Cholesterol-modified siCREB5 and siTOP1MT were used to silence the expression of CREB5 and TOP1MT in the xenograft tumor models. The results showed that compared with that in the control group, tumor weights in the gene silencing groups were significantly reduced. Among them, the double-targeting group (siCREB5 and siTOP1MT) resulted in the most obvious inhibition of tumor weight (Fig. 6A). Similarly, tumor growth rates in the siTOP1MT group, siCREB5 group, and siCREB5+siTOP1MT group were significantly reduced, while the combination group had the best response to cisplatin treatment (Fig. 6B).

Moreover, the synergistic inhibition of TOP1MT and CREB5 could dramatically decrease the positive percentage of Ki67 (Fig. 6C). These results showed that double-targeting of TOP1MT and CREB5 had a potent antitumor effect in the cisplatin-resistant model. To confirm the gene silencing effect in vivo, the tumor tissues were analyzed by RT-PCR and immunoblotting assay. The mRNA and protein levels of TOP1MT and CREB5 were significantly suppressed, especially in the double-targeting group (Fig. 6D, E).

To evaluate the therapeutic value of siCREB5 and siTOP1MT in HNSCC in vivo, we constructed the PDX model with cisplatin-resistant patient samples (Fig. 6F, G). The results showed that siCREB5 and siTOP1MT significantly inhibited the growth of HNSCC PDXs in vivo (Fig. 6H, I)*.* The TGI was 82.35%. The tumor weight was significantly decreased in the treatment group compared with the control group (Fig. 6J). These results indicated that double-targeting of CREB5 and TOP1MT had a significant effect in overcoming the cisplatin resistance of HNSCC.

### CREB5 positively correlates with TOP1MT expression in HNSCC patients

**Fig. 7:**
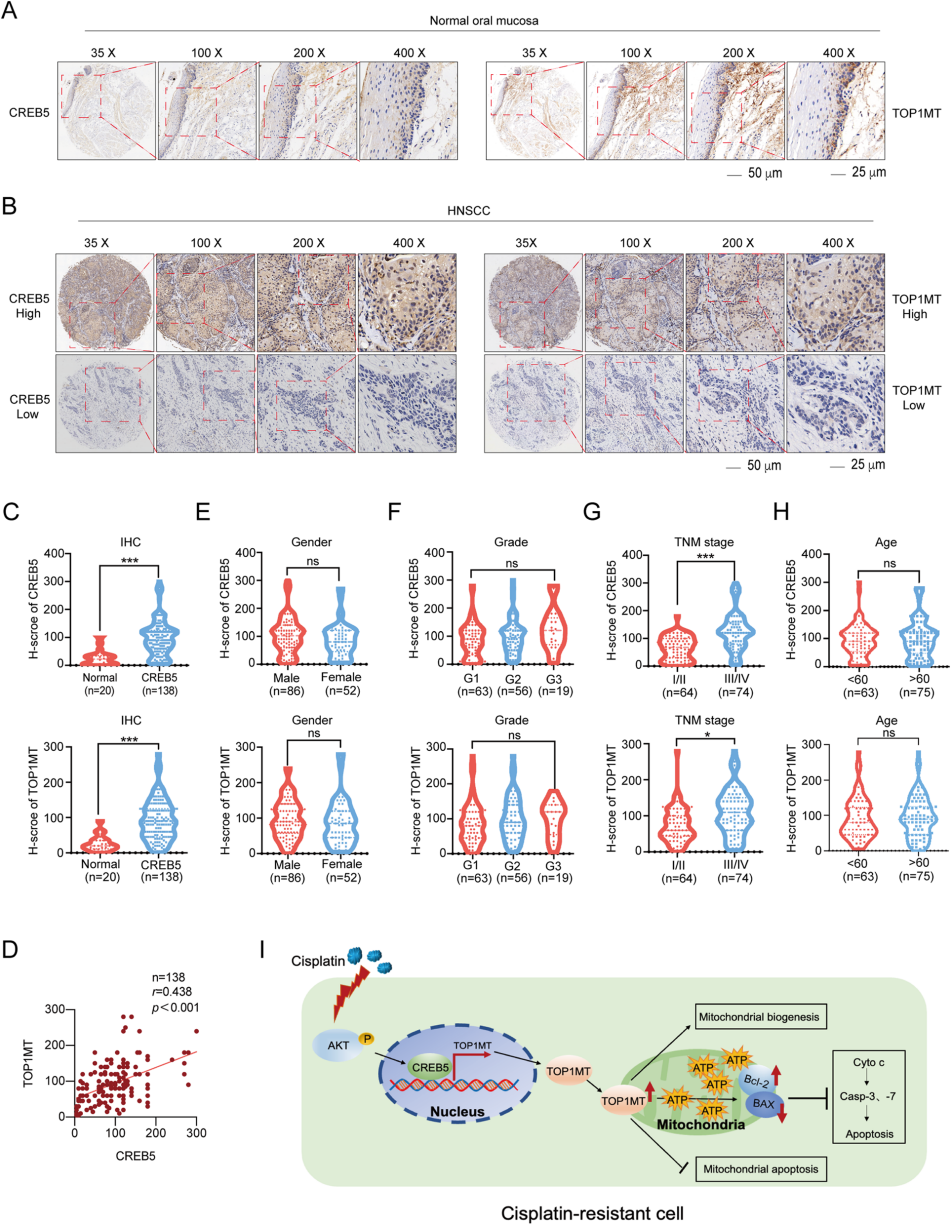
A positive correlation between CREB5 and TOP1MT expression was observed in HNSCC tissues. **A** CREB5 and TOP1MT expression was detected using IHC in normal oral tissues. **B** Representative images of CREB5 and TOP1MT expression in HNSCC tissues. **C** The expression scores of CREB5 and TOP1MT were analyzed in normal and HNSCC tissues. **D** IHC analysis of the correlation between CREB5 and TOP1MT expression in the HNSCC tissue microarray. **E**–**H** The correlations between the expression scores of TOP1MT and CREB5 and gender, grade, TNM stage, and age were analyzed in normal and HNSCC tissues. **I** The proposed model illustrates the regulatory role of TOP1MT and CREB5 in promoting cisplatin resistance in HNSCC. *p < 0.05; **p < 0.01; ***p < 0.001

To further explore the correlation between CREB5 and TOP1MT, immunohistochemistry was performed in an HNSCC tissue microarray. First, we found that both CREB5 and TOP1MT were significantly higher in tumor tissues than in the normal control (Fig. 7A–C). Moreover, there was a positive correlation between the expressions of CREB5 and TOP1MT (*r* = 0.438, Fig. 7D). In addition, we analyzed the correlation between protein expression and clinical characteristics and found that higher expression of CREB5 and TOP1MT correlated with the advanced TNM stage, while not with others **(**Fig. 7E–H).

Overall, this study demonstrated that CREB5 nucleus translocation mediated by AKT phosphorylation induced by cisplatin resistance transcriptionally activated TOP1MT, which inhibited mitochondrial apoptosis through Bcl-2 upregulation and Bax downregulation in HNSCC (Fig. 7I).

## Discussion

Cisplatin is a common clinical antitumor drug that is widely used in diseases including HNSCC, lung cancer, and lymphoma [30, 31]. Its discovery and application are of great significance to the development of tumor chemotherapy. However, intrinsic or acquired drug resistance severely limits the clinical application of cisplatin [32]. Although the mechanisms of cisplatin resistance have been partially discovered, the clinical status of cancer patients with cisplatin resistance has not improved. It is undeniable that most alternatives are not as effective as cisplatin. For example, carboplatin can replace cisplatin as a chemotherapy drug in patients with renal insufficiency, but its efficacy is not as good as cisplatin [33]. Therefore, it is urgent to deeply explore the molecular mechanism of cisplatin resistance and formulate appropriate anti-cisplatin resistance strategies. Here, we provide a fundamental basis for the use of a double-targeting strategy with CREB5 and TOP1MT to overcome cisplatin resistance in HNSCC.

CREB5 is a member of the CRE-BP1 family of the cAMP response element-binding proteins [34, 35]. To date, studies on the role of CREB5 in tumors have mainly focused on invasion, metastasis, and proliferation [12, 16, 36–40]. In addition, it has been reported that CREB5 expression can negatively regulate HBV and human enterovirus-a71 (EV-a71) replication [41, 42]. CREB5 was also found as a transcription factor to regulate prg4 expression and prevent arthritis [43]. As a diagnostic marker, CREB5 is valuable in assessing the prognosis of cancer patients. Bo Deng et al. found that high expression of CREB5, PTPRB, and COL4A3 could predict disease-free survival in lung cancer [44]. Huan Song et al. established a prognostic management model based on the characteristics of seven genes (CREB5, etc.) in breast cancer, which showed that patients with high risk score had a poor prognosis [45].

Furthermore, there are four related literatures on the PubMed website reporting the relationship between CREB5 and tumor resistance [17, 46–48], of which only one paper preliminarily clarified the molecular mechanism of CREB5 promoting prostate cancer resistance to androgen receptor antagonists [17]. In this study, we identified for the first time that CREB5 confers cisplatin resistance and regulates mitochondrial apoptosis in HNSCC. Our data confirm that cisplatin-induced phosphorylation of AKT could promote CREB5 entry into the nucleus, and TOP1MT was identified as a novel transcriptional target of CREB5. TOP1MT plays an indispensable role in cisplatin resistance and mitochondrial apoptosis mediated by CREB5.

TOP1MT, encoded by a nuclear gene, is the only topoisomerase located in mitochondria due to its N-terminal mitochondrial targeting sequence [49, 50]. TOP1MT plays an indispensable role in reducing the tension of mtDNA replication and transcription and maintains the integrity of mtDNA [28]. TOP1MT affects mitochondrial function, ATP production, and apoptosis by regulating mtDNA transcription, replication, and translation [51–53]. Studies have shown that TOP1MT promotes tumor growth by enhancing the synthesis of mitochondrial-related proteins [51, 54]. In a liver regeneration model, researchers found that a lack of TOP1MT reduced the proliferation of hepatocytes by limiting the amplification of mtDNA [28, 55]. This result shows that TOP1MT plays an important role in maintaining cell homeostasis. In addition, Wang et al. found that TOP1MT deficiency enhanced glucose aerobic glycolysis by stimulating LDHA to promote GC progression [56].

However, the mechanisms of abnormal expression of TOP1MT are unclear. Only one study has shown that MYC may be a factor regulating TOP1MT expression [57]. In this study, we describe in detail the regulation process of the whole cisplatin-Akt-CREB5-TOP1MT axis. Cisplatin induces the activation of AKT signaling pathway in HNSCC cells, and the activated AKT signaling pathway promotes the nuclear translocation of CREB5. As a transcription factor, CREB5 significantly promotes the transcription of the TOP1MT gene. Our study provides new insights into the mechanism of aberrant expression of TOP1MT.

In general, our study elucidated that CREB5 increased cell mitochondrial activity and ATP production through TOP1MT, promoted the expression of the anti-apoptotic protein Bcl-2, and enhanced the Bcl-2/Bax ratio, thereby inhibiting the mitochondrial apoptosis pathway and promoting HNSCC cell resistance to cisplatin. In addition, the results of subcutaneous tumor-bearing experiments in nude mice indicate that treatments targeting CREB5 or TOP1MT have a therapeutic effect on HNSCC. More excitingly, double-targeting of CREB5 and TOP1MT showed a powerful antitumor effect, which would be a promising strategy for combating cisplatin resistance.

## Conclusions

In summary, our study demonstrated that the novel CREB5/TOP1MT axis promotes cisplatin resistance in HNSCC cells through inhibiting mitochondrial apoptosis, and this double-targeting therapy provides a new avenue for treating cisplatin-resistant patients.

## Supplementary Information

**Additional file 1: Fig. S1**. The quantification of immunoblotting images was analyzed by image J. **Fig. S2**. Immunoblotting analysis of AKT and nuclear CREB5 expression after down regulated AKT expression. **Fig. S3**. The positive percentage of Ki67 increased in CREB5 overexpression tissues. **Fig. S4**. The quantification of immunoblotting images was analyzed by image J. **Fig. S5**. RT-PCR was used to detect the effect of CREB5 on the expression of mitochondrial-related gene in HN4 and HN30 cells. **Fig. S6**. Immunoblotting analysis of TOP1MT expression in untreated or KG-501 treated or KG-501/MK2206 treated cisplatin-resistant cells. **Fig. S7**. The quantification of immunoblotting images was analyzed by image J. **Fig. S8**. Cisplatin resistance enhances mitochondrial activity of HNSCC cells. **Fig. S9**. CREB5 regulates mitochondrial activity through TOP1MT. **Fig. S10**. The quantification of immunoblotting images was analyzed by image J. **Table S1**. Primary antibodies used in this study. **Table S2**. The primers used for real-time PCR analysis. **Table S3**. The small interfering RNA (siRNA) sequences used in this study. **Table S4**. Sequences of ChIP-PCR primers among TOP1MT promoter region.

**Additional file 2.** The images of the original, uncropped gels/blots.

## Data Availability

All data supporting this study are available within this article and supplementary information file. The data about the Agilent expression profile chip experiment have been deposited into the Gene Expression Omnibus repository under accession number GSE202030. You may view the GSE202030 study at https://www.ncbi.nlm.nih.gov/geo/query/acc.cgi?acc=GSE202030. The dataset used and/or analyzed during the current study is available from the corresponding author on reasonable request.

## Abbreviations

ChIP: Chromatin immunoprecipitation
CREB: cAMP response element-binding
CREB5: cAMP response element-binding 5
CR-HNSCC: Cisplatin-resistant HNSCC
DDP: Cis-Diammin-odichloroplatinum II dichloride
HNSCC: Head and neck cancer
IC50: Half maximal inhibitory concentration
IHC: Immunohistochemical
KEGG: Kyoto Encyclopedia of Genes and Genomes
MT-ATP: Measurement of mitochondrial-ATP
mtDNA: Mitochondrial DNA
NRF: Nuclear respiratory factor
OCR: Oxygen consumption rate
PDX: Patient-derived xenograft
PGC-1α: Peroxisome proliferator-activated receptor gamma coactivator 1-alpha
RT-PCR: Real-time PCR
TFAM: Mitochondrial transcription factor A
TMA: Tissue microarray
TNM: Tumor Node Metastasis
TOP1MT: DNA topoisomerase I mitochondria
TPF: Docetaxel, cisplatin, 5-FU
