# Supplementary material for: A novel CREB5/TOP1MT axis confers cisplatin resistance through inhibiting mitochondrial apoptosis in head and neck squamous cell carcinoma
Source: BMC Med. 2022 Jul 1;20:231. doi: 10.1186/s12916-022-02409-x (PMC9248137; doi:10.1186/s12916-022-02409-x)
Supplement: Supplementary file 1 — Additional file 1: Fig. S1. The quantification of immunoblotting images was analyzed by image J. Fig. S2. Immunoblotting analysis of AKT and nuclear CREB5 expression after down regulated AKT expression. Fig. S3. The positive percentage of Ki67 increased in CREB5 overexpression tissues. Fig. S4. The quantification of immunoblotting images was analyzed by image J. Fig. S5. RT-PCR was used to detect the effect of CREB5 on the expression of mitochondrial-related gene in HN4 and HN30 cells. Fig. S6. Immunoblotting analysis of TOP1MT expression in untreated or KG-501 treated or KG-501/MK2206 treated cisplatin-resistant cells. Fig. S7. The quantification of immunoblotting images was analyzed by image J. Fig. S8. Cisplatin resistance enhances mitochondrial activity of HNSCC cells. Fig. S9. CREB5 regulates mitochondrial activity through TOP1MT. Fig. S10. The quantification of immunoblotting images was analyzed by image J. Table S1. Primary antibodies used in this study. Table S2. The primers used for real-time PCR analysis. Table S3. The small interfering RNA (siRNA) sequences used in this study. Table S4. Sequences of ChIP-PCR primers among TOP1MT promoter region. [file 12916_2022_2409_MOESM1_ESM.docx]

**Supplementary Materials for**

**A novel CREB5/TOP1MT axis confers cisplatin resistance through inhibiting mitochondrial apoptosis in head and neck squamous cell carcinoma**

Tong Tong^1,2,3,#^, Xing Qin^1,#^, Yingying Jiang^4^, Haiyan Guo^5^, Xiaoning Wang^6^, Yan Li^7^, Fei Xie^1^, Hao Lu^1^, Peisong Zhai^1^, Hailong Ma^1,8*^ and Jianjun Zhang^1,8*^

*** Corresponding Authors:**

Jianjun Zhang & Hailong Ma

M.D., Ph.D.

Department of Oral and Maxillofacial-Head & Neck Oncology,

Shanghai Ninth People’s Hospital, Shanghai Jiao Tong University School of Medicine, No. 639, Zhizaoju Rd, Shanghai 200011, PR China;

Tel.: (86) 21-23271699-4239;

E-mail address: [zjjbio@sjtu.edu.cn](mailto:zjjbio@sjtu.edu.cn)

[mahl21@sjtu.edu.cn](mailto:mahl21@sjtu.edu.cn)

**Supplementary data: 10 figures, 4 tables**

**Additional file: Supplementary figures**

**
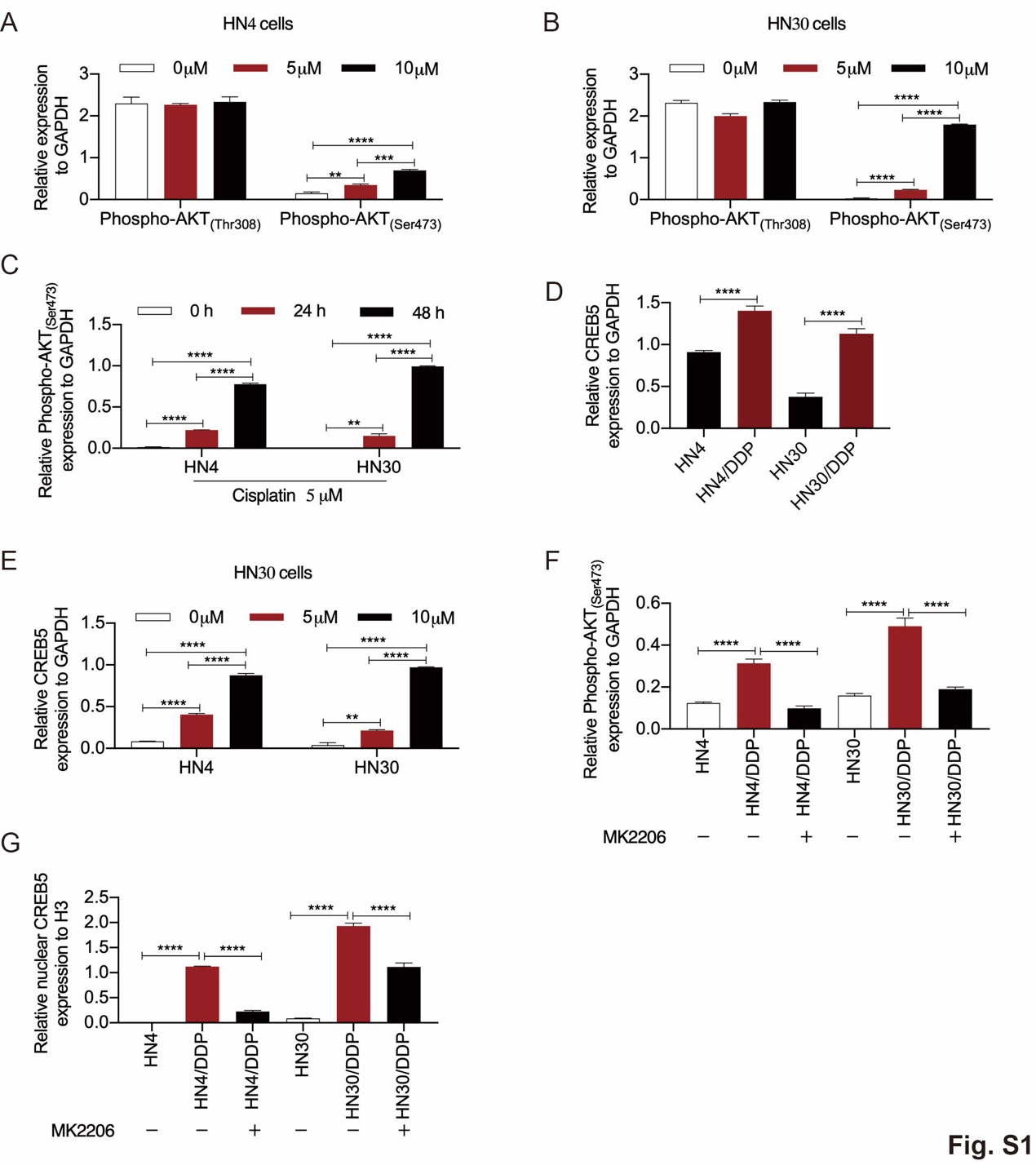
**

**Fig. S1.** The quantification of immunoblotting images was analyzed by image J. ***p* < 0.01; ****p* < 0.001; *****p* < 0.0001.

**
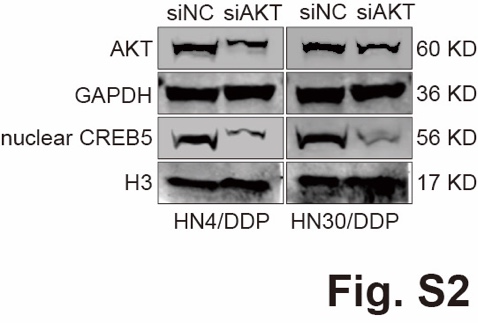
**

**Fig. S2.** Immunoblotting analysis of AKT and nuclear CREB5 expression after down regulated AKT expression.

**
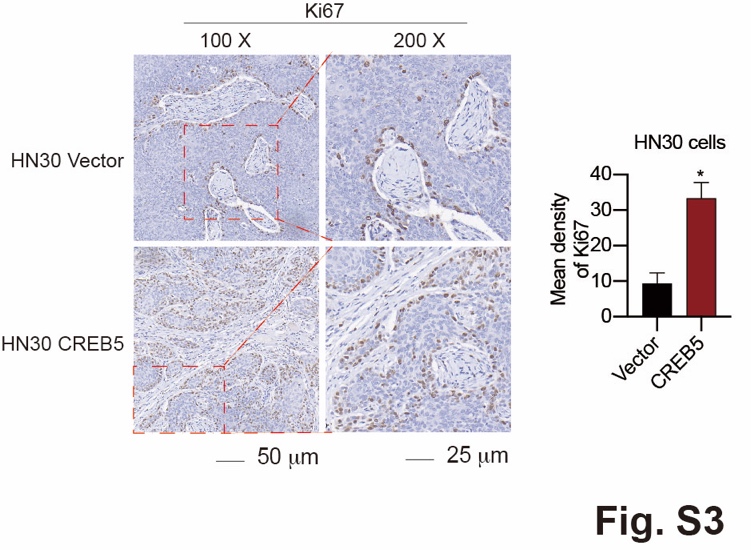
**

**Fig. S3.** The positive percentage of Ki67 increased in CREB5 overexpression tissues. Immunohistochemical (IHC) staining image showing Ki67 in subcutaneous tumor tissue of nude mice. The histogram shows the quantitative analysis of IHC. **p* < 0.05.


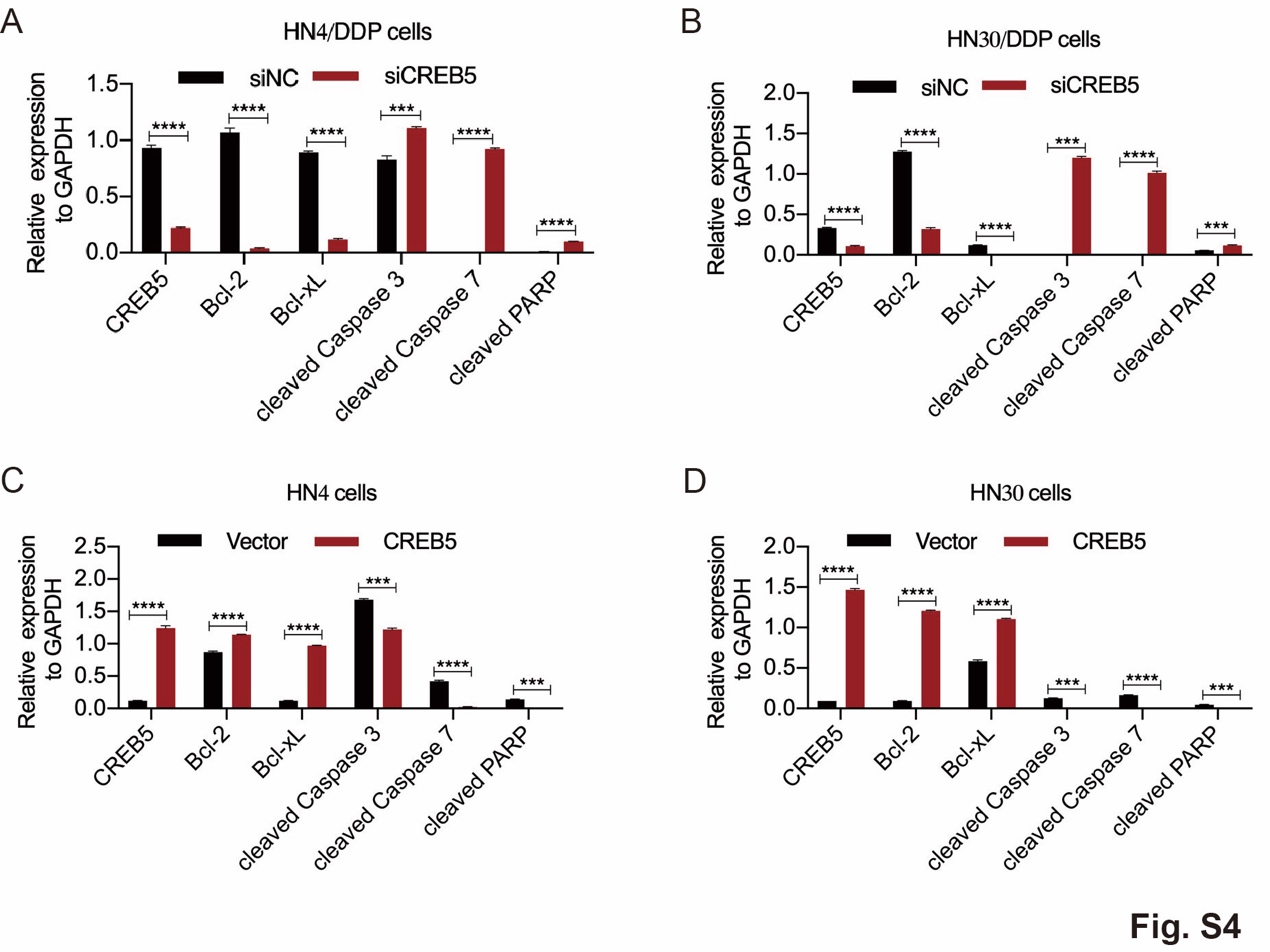


**Fig. S4.** The quantification of immunoblotting images was analyzed by image J. ****p* < 0.001; *****p* < 0.0001.


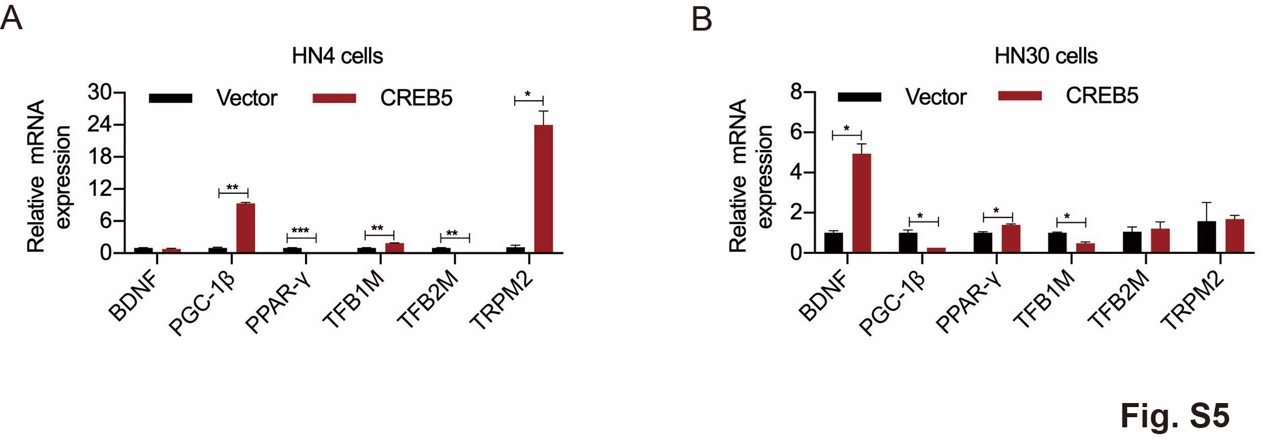


**Fig. S5.** RT-PCR was used to detect the effect of CREB5 on the expression of mitochondrial-related gene in HN4 and HN30 cells. **p* < 0.05; ***p* < 0.01; ****p* < 0.001.


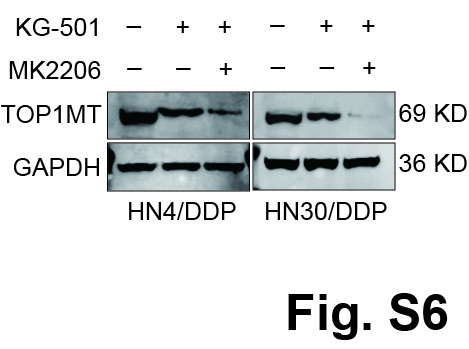


**Fig. S6.** Immunoblotting analysis of TOP1MT expression in untreated or KG-501 treated or KG-501/MK2206 treated cisplatin-resistant cells.

**
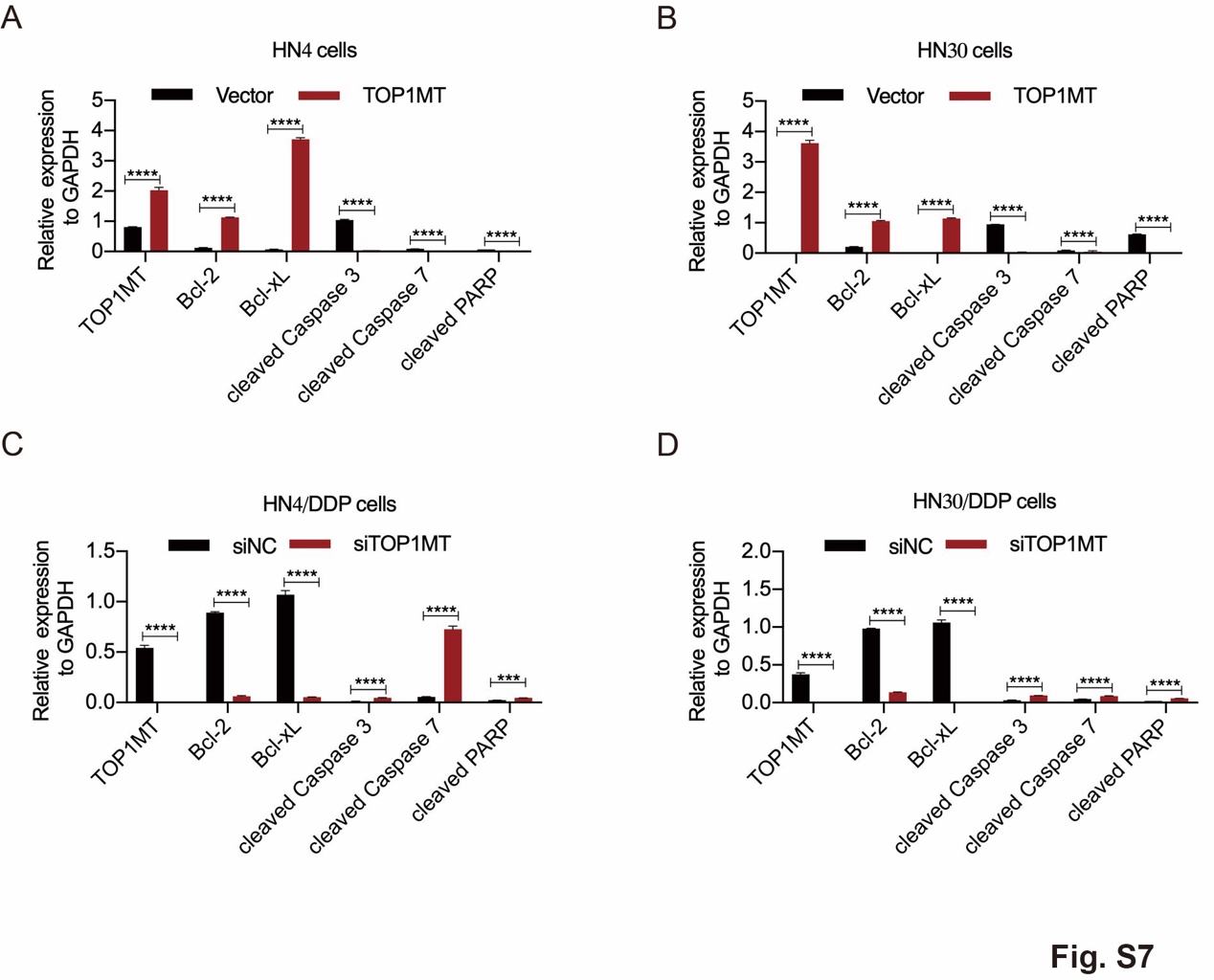
**

**Fig. S7.** The quantification of immunoblotting images was analyzed by image J. ****p* < 0.001; *****p* < 0.0001.

**
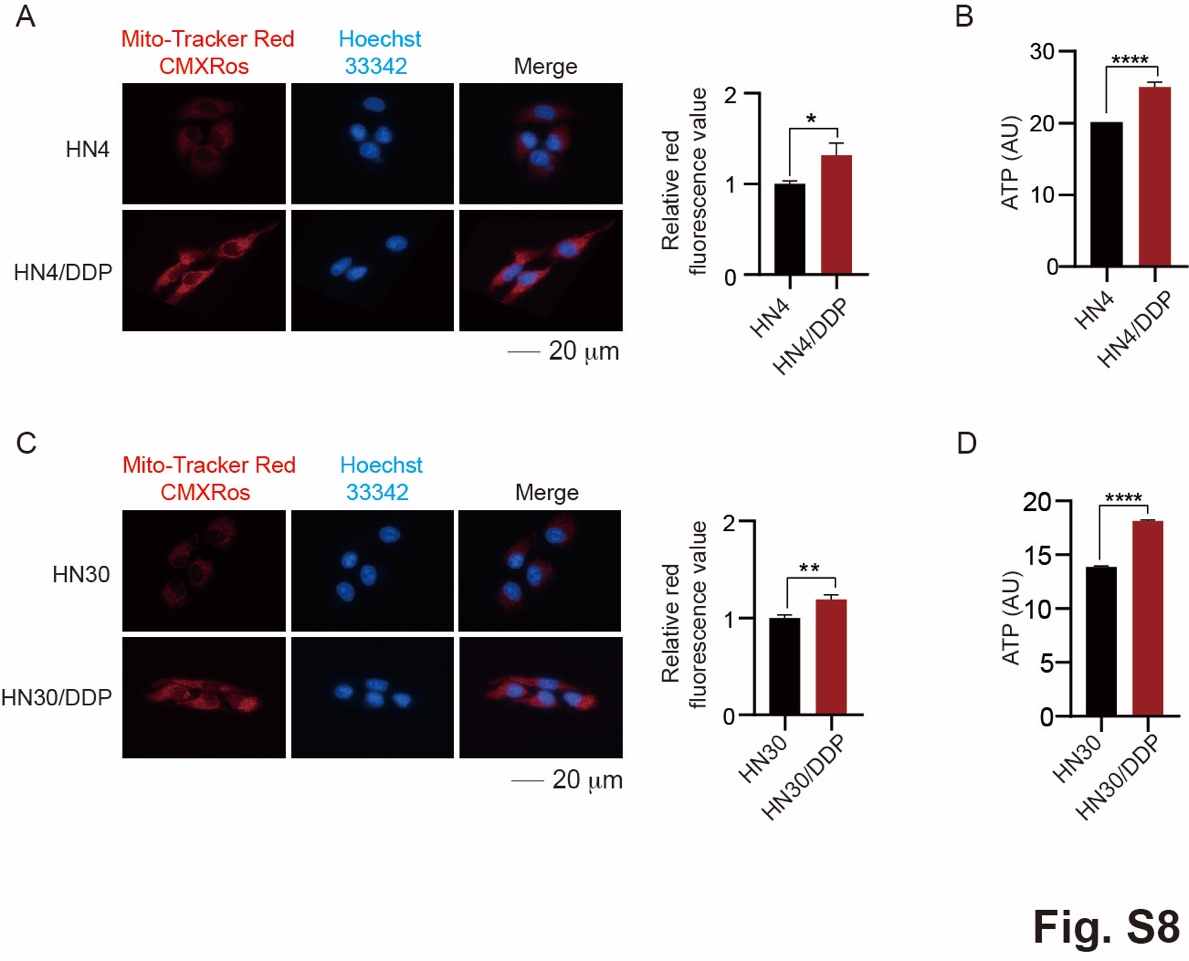
**

**Fig. S8.** Cisplatin resistance enhances mitochondrial activity of HNSCC cells. (A) Mito-Tracker Red CMXRos is used to detect mitochondrial activity in HN4 and HN4/DDP cells. Relative red fluorescence values are shown on the right. (B) The ATP level is detected in HN4 and HN4/DDP cells. (C) Mito-Tracker Red CMXRos is used to detect mitochondrial activity in HN30 and HN30/DDP cells. Relative red fluorescence values are shown on the right. (D) The ATP level is detected in HN30 and HN30/DDP cells. **p* < 0.05; ***p* < 0.01; *****p* < 0.0001.

**
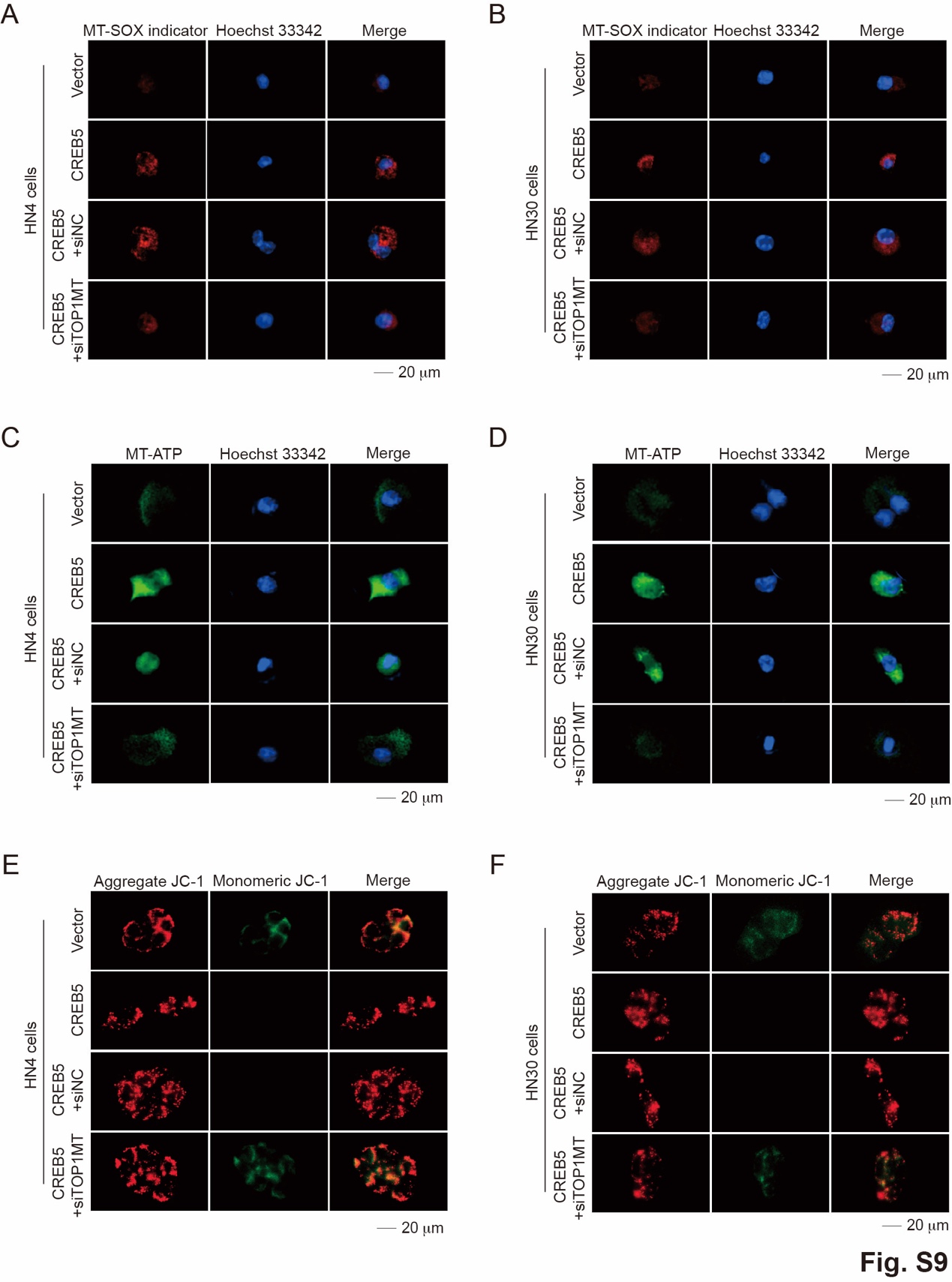
**

**Fig. S9.** CREB5 regulates mitochondrial activity through TOP1MT. (A, B) MitoSOX Red Mitochondrial Superoxide Indicator (MT-ROS indicator) was used to detect the effect of CREB5 and TOP1MT expression on mitochondrial-ROS. (C, D) Mitochondrial-ATP (MT-ATP) fluorescent probe pCMV-Mito-AT1.03 was used to detect the effect of CREB5 and TOP1MT expression on mitochondrial ATP. (E, F) Enhanced mitochondrial membrane potential assay kit with JC-1 was used to detect the effect of CREB5 and TOP1MT expression on mitochondrial membrane protein. Normal mitochondrial potential showed red fluorescence (aggregate JC-1), and damaged mitochondrial potential showed green fluorescence (monomeric JC-1).

**
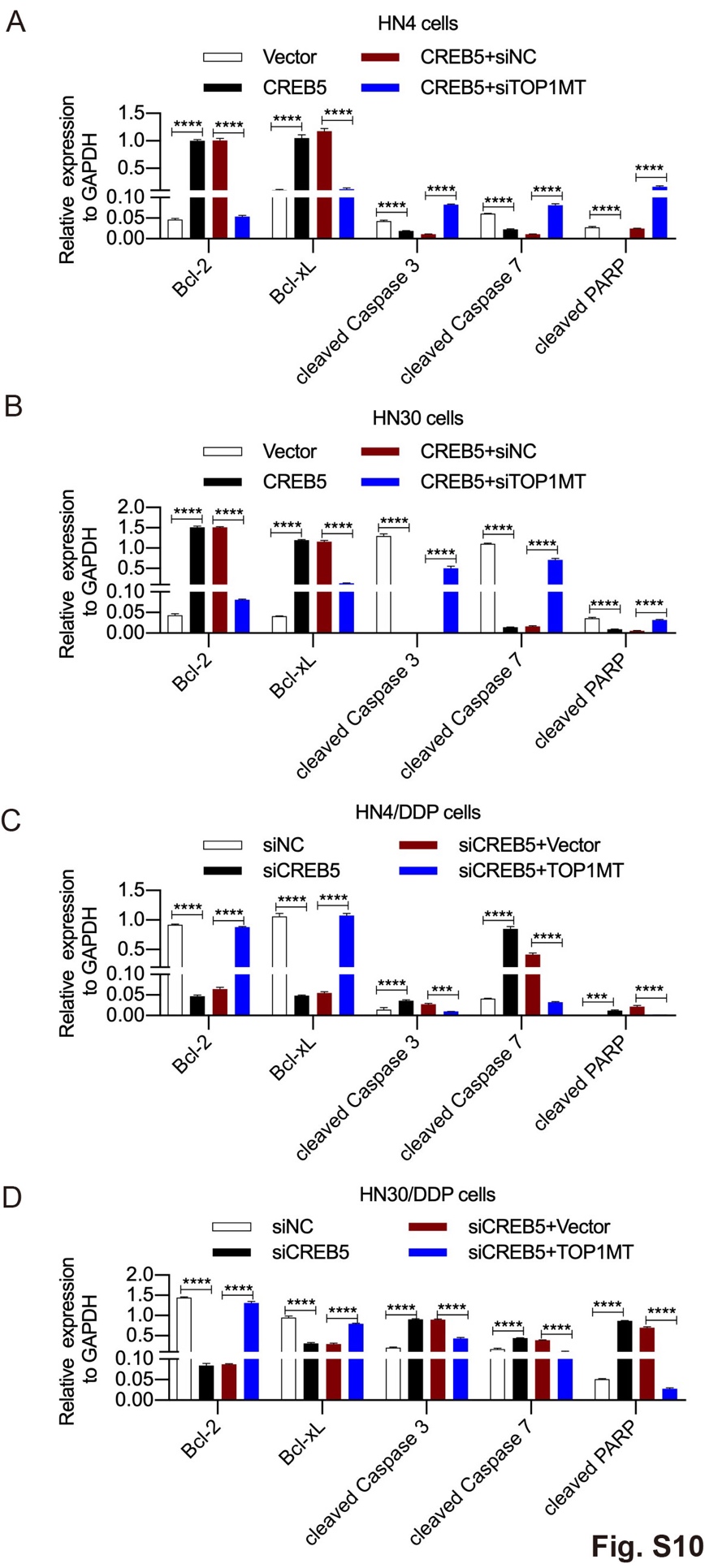
**

**Fig. S10.** The quantification of immunoblotting images was analyzed by image J. ****p* < 0.001; *****p* < 0.0001.

**Additional file: Supplementary tables**

**Table S1. Primary antibodies used in this study.**

| **Antigens** | **Manufacturer** | **Application** |
| --- | --- | --- |
| TOP1MT | Novus (Cat #NBP2-44360) | 1:100 for WB  1:200 for IHC |
| CREB5 | Proteintech (Cat #14196-1-AP, RRID:AB_2083832) | 1:1,000 for WB |
|  | ABclonal (Cat #A14635, RRID:AB_2761510) | 1:100 for ChIP  1:200 for HE |
| Cytochrome C | Proteintech (Cat #10993-1-AP, RRID:AB_2090467) | 1:1,000 for WB |
| Caspase 7 | Cell Signaling Technology (Cat #9492, RRID:AB_2228313) | 1:1,000 for WB |
| Caspase 3 | Proteintech (Cat #19677-1-AP, RRID:AB_10733244) | 1:1,000 for WB |
| PARP | Cell Signaling Technology (Cat #9532, RRID:AB_659884) | 1:1,000 for WB |
| NRF1 | Proteintech (Cat #12482-1-AP, RRID:AB_2282876) | 1:1,000 for WB |
| NRF2 | Proteintech (Cat #16396-1-AP, RRID:AB_2782956) | 1:1,000 for WB |
| TFAM | Proteintech (Cat #19998-1-AP) | 1:1,000 for WB |
| AKT | Cell Signaling Technology (Cat #4691, RRID:AB_915783) | 1:1,000 for WB |
| Phospho-AKT  (Ser 473) | Cell Signaling Technology (Cat #4060, RRID:AB_2315049) | 1:1,000 for WB |
| Phospho-AKT  (Thr 308) | Cell Signaling Technology (Cat #13038) | 1:1,000 for WB |
| Bcl-2 | Proteintech (Cat #12789-1-AP) | 1:500 for WB |
| Bcl-xL | ABclonal (Cat #A19703, RRID:AB_2862745) | 1:500 for WB |
| Bax | Proteintech (Cat #50599-2-Ig, RRID:AB_2061561) | 1:1,000 for WB |
| AMPK | Abcam (Cat #ab32047, RRID:AB_722764) | 1:1,000 for WB |
| Phospho-AMPK | Abcam (Cat #ab133448) | 1:1,000 for WB |
| Histone 3 | Cell Signaling Technology (Cat#4499, RRID:AB_10544537) | 1:2,000 for WB |
| GAPDH | Proteintech (Cat #60004-1-Ig) | 1:10,000 for WB |
| FLAG | Proteintech (Cat #20543-1-AP, RRID:AB_11232216) | 1:1,000 for WB |

Abbreviation: WB, Immunoblotting; ChIP, Chromatin immunoprecipitation assay; IHC, Immunohistochemistry.

**Table S2. The primers used for real-time PCR analysis.**

| **Gene** | **Primer sequences** |
| --- | --- |
| Human *TOP1MT* | forward 5’-CGGTGGCCCTGTATTTCAT-3' |
|  | reverse 5’-GCCTCACCGTCCTCCCTC-3’ |
| Human *CREB5* | forward 5’-ATTGACTCACCACCCTGCTG-3' |
|  | reverse 5’-GCATGAAGGTGGGAATGGGA-3’ |
| Human *NRF1* | forward 5’-GCTGATGAAGACTCGCCTTCT-3' |
|  | reverse 5’-TACATGAGGCCGTTTCCGTTT-3’ |
| Human *NRF2* | forward 5’-TCAGCGACGGAAAGAGTATGA-3' |
|  | reverse 5’-CCACTGGTTTCTGACTGGATGT-3’ |
| Human *TFAM* | forward 5’-ATGGCGTTTCTCCGAAGCAT-3' |
|  | reverse 5’-TCCGCCCTATAAGCATCTTGA-3’ |
| Human *PGC-1α* | forward 5’-TCTGAGTCTGTATGGAGTGACAT-3' |
|  | reverse 5’-CCAAGTCGTTCACATCTAGTTCA-3’ |
| Human *CDKN1A* | forward 5’-CGATGGAACTTCGACTTTGTCA-3' |
|  | reverse 5’-GCACAAGGGTACAAGACAGTG-3’ |
| Human *CCND2* | forward 5’-TTTGCCATGTACCCACCGTC-3' |
|  | reverse 5’-AGGGCATCACAAGTGAGCG-3’ |
| Human *NOS3* | forward 5’-TGATGGCGAAGCGAGTGAAG-3' |
|  | reverse 5’-ACTCATCCATACACAGGACCC-3’ |
| Human *EGF* | forward 5’-TGGATGTGCTTGATAAGCGG-3' |
|  | reverse 5’-ACCATGTCCTTTCCAGTGTG-3’ |
| Human *JAK3* | forward 5’-CCTGATCGTGGTCCAGAGAG -3' |
|  | reverse 5’-GCAGGGATCTTGTGAAATGTCAT-3’ |
| Human *TFB1M* | forward 5’-GTTGCCCACGATTCGAGAAAT-3' |
|  | reverse 5’-GCCCACTTCGTAAACATAAGCAT-3’ |
| Human *TFB2M* | forward 5’-CCAAGGAAGGCGTCTAAGGC-3' |
|  | reverse 5’-CTTTCGAGCGCAACCACTTTG-3’ |
| Human *TRPM2* | forward 5’-TTCGTGGATTCCTGAAAACATCA-3' |
|  | reverse 5’-CCAGCATCAGACAGTTTGGAAC-3’ |
| Human *BNDF* | forward 5’-GGCTTGACATCATTGGCTGAC-3' |
|  | reverse 5’-CATTGGGCCGAACTTTCTGGT-3’ |
| Human *PPAR-γ* | forward 5’-TACTGTCGGTTTCAGAAATGCC-3' |
|  | reverse 5’-GTCAGCGGACTCTGGATTCAG-3’ |
| Human *PGC-1β* | forward 5’-GATGCCAGCGACTTTGACTC-3' |
|  | reverse 5’-ACCCACGTCATCTTCAGGGA-3’ |
| Human *β-actin* | forward 5’-TCACCCACACTGTGCCCATCTACGA -3' |
|  | reverse 5’-CAGCGGAACCGCTCATTGCCAATGG -3’ |
| Human *GAPDH* | forward 5’-GGAGCGAGATCCCTCCAAAAT-3' |
|  | reverse 5’-GGCTGTTGTCATACTTCTCATGG -3’ |

**Table S3. The small interfering RNA (siRNA) sequences used in this study.**

| **Name** | **siRNA sequence** |
| --- | --- |
| siRNA-NC | Sense 5’-UAAGGCUAUGAAGAGAUACdTdT-3’ |
|  | Antisense 5’-GUAUCUCUUCAUAGCCUUAdTdT-3’ |
| siRNA-TOP1MT | Sense 5’-GGAAGAACUUCUUCAAUGACU-3’ |
|  | Antisense 5’-AGUCAUUGAAGAAGUUCUUCC-3’ |
| siRNA-CREB5 | Sense 5’-GCGGAAUAUCUCGAUGCAUdTdT-3’ |
|  | Antisense 5’-AUGCAUCGAGAUAUUCCGCdTdT-3’ |
| siRNA-AKT | Sense 5’-GGAGAUCAUGCAGCAUCGCdTdT-3’ |
|  | Antisense 5’-GCGAUGCUGCAUGAUCUCCdTdT-3’ |

**Table S4. Sequences of ChIP-PCR primers among TOP1MT promoter region.**

| **No.** | **Primer sequences** |
| --- | --- |
| Primer 1 | forward 5’- GTCAACTGAGGCCAAGGCTTT -3' |
|  | reverse 5’- CATTACCGCTCAGTCACCGA-3’ |
| Primer 2 | forward 5’- CTGAGCGGTAATGGTGTTGTC-3' |
|  | reverse 5’- TTTCCTGTTGTGGTGAGTAATGG-3’ |
| Primer 3 | forward 5’- CTGCCTATGTCCATTTGTTAAGAAG-3' |
|  | reverse 5’- CAGGCTTCTAGTTTGGGTGGTC-3’ |
| Primer 4 | forward 5’- ATCCTCAACTCGCTCATTCTCA-3' |
|  | reverse 5’- TCAGCCGCACTGCATGTGT-3’ |
| Primer 5 | forward 5’- ACACATGCAGTGCGGCTGA-3' |
|  | reverse 5’- ACAGTTCCGAGTGGGTTTGCT-3’ |
| Primer 6 | forward 5’- CCACTCGGAACTGTGTCCTGT-3' |
|  | reverse 5’- AGTCTGGAAAATTGAGCGGATG-3’ |
| Primer 7 | forward 5’- GCCACATCCGCTCAATTTTC-3' |
|  | reverse 5’- CCTGAGGTGCAAGCTGTCAC-3’ |
| Primer 8 | forward 5’- TCTGGGATCTCACGTCACCC-3' |
|  | reverse 5’- CGCTTCTGCGACTCTGAAGAT-3’ |
